# First-line Immuno-chemotherapy for extensive-stage small-cell lung cancer: A network meta-analysis and cost-effectiveness analysis

**DOI:** 10.3389/fpubh.2023.1028202

**Published:** 2023-03-16

**Authors:** Youwen Zhu, Kun Liu, Qiuping Yang, Manting Zeng, Libo Peng

**Affiliations:** ^1^Department of Oncology, Xiangya Hospital, Central South University, Changsha, Hunan, China; ^2^Department of Pathology, Tangshan Cancer Hospital, Tangshan, Hebei, China; ^3^National Clinical Research Center for Geriatric Disorders, Xiangya Hospital, Central South University, Changsha, Hunan, China; ^4^Department of Oncology, Loudi Central Hospital, Loudi, Hunan, China

**Keywords:** extensive-stage small-cell lung cancer, immuno-chemotherapy, network meta-analysis, cost-effectiveness, quality-adjusted life-years

## Abstract

**Introduction:**

Many randomized controlled trials have indicated that immuno-chemotherapy could generate clinical benefits, though the cost of immuno-chemotherapy was so prohibitive and the options were varied. This investigation aimed at evaluating effectiveness, safety, and cost-effectiveness for immuno-chemotherapy as a first-line therapeutic option for ES-SCLC patients.

**Methods:**

Multiple scientific literature repositories were searched for clinical studies where immuno-chemotherapy was regarded as the first-line treatment for ES-SCLC, which were published in English between Jan 1, 2000, and Nov 30, 2021. This study conducted a network meta-analysis (NMA) and cost-effectiveness analysis (CEA) based upon US-resident payer perspectives. Overall survival (OS), progression-free survival (PFS), and adverse events (AEs) were evaluated through NMA. In addition, costings, life-years (LYs), quality-adjusted life-years (QALYs), and incremental cost–benefit ratio (ICER) were estimated by CEA.

**Results:**

We identified 200 relevant search records, of which four randomized controlled trials (RCTs) (2,793 patients) were included. NMA demonstrated that the effect of atezolizumab plus chemotherapy was ranked at a more elevated position in comparison to other immuno-chemotherapy options and chemotherapy alone, within the general population. The influence of atezolizumab plus chemotherapy and durvalumab plus chemotherapy was ranked higher within populations experiencing non-brain metastases (NBMs) andbrain metastases (BMs), respectively. The CEA revealed that the ICERs of immuno-chemotherapy over chemotherapyalone were higher than the willingness-to-pay (WTP) threshold of $150,000/QALY in any population. However, treatment with atezolizumab plus chemotherapy and durvalumab plus chemotherapy were more favorable health advantages than other immuno-chemotherapy regimens and chemotherapy alone, and the results were 1.02 QALYs and 0.89 QALYs within overall populations and populations with BMs, respectively.

**Conclusion:**

The NMA and cost-effectiveness investigation demonstrated that atezolizumab plus chemotherapy could be an optimal first-line therapeutic option for ES-SCLC when compared with other immuno-chemotherapy regimens. Durvalumab plus chemotherapy is likely to be the most favorable first-line therapeutic option for ES-SCLC with BMs.

## 1. Introduction

Lung cancer has the second-highest morbidity and highest mortalityamong all cancer models globally, with over 2.2 million and 230,000 cases diagnosed, and over 1.79 million and 130,000 deaths occurring globally and within the United States (US) in 2021, respectively (1, 2). Small cell lung cancer accounted for more than 10% of lung cancer, and up to 60% were diagnosed as extensive-stage small cell lung cancer (ES-SCLC), with a 5-year survival rate of only 2% (3–5). The most common distant metastases were brain metastases (BMs), which are prevalent within 10% of such clinical cases at initial diagnosis, accounting for more than 50% incidence within 2 years (6).

During the past 30 years, etoposide plus platinum (EP) was established as a first-line chemotherapeutic option for ES-SCLC, though the survival of patients has not improved significantly, and patients typically endure recurrence within 1–2 years. A phase III clinical data of ES-SCLC demonstrated that the survival time of the chemotherapeutics group increased by only 0.63 days per year (7). Therefore, it is necessary and urgent to develop new drugs to treat ES-SCLC.

The wide use of immune checkpoint inhibitors (ICIs) has paved the road for a novel age of oncology therapeutics, which could block the programmed cell death 1 (PD-1), programmed death-ligand 1(PD-L1), and cytotoxic T lymphocyte-associated protein 4 (CTLA-4) signaling pathways, and are becoming a novel treatment for ES-SCLC since such schemes could enhance survival rate and quality-of-life. For example, the IMpower133 study demonstrated that adding atezolizumab (PD-L1) to chemotherapy for first-line treatment of ES-SCLC resulted in significant improvement in overall survival (OS, hazard ratio [HR], 0.76; 95% confidence interval [CI], 0.60 to 0.95; *p* = 0.0154) and progression-free survival (PFS, HR, 0.77; 95% CI, 0.62 to 0.96; *p* = 0.02) versus chemotherapy (8, 9). The CASPIAN study showed that it sustained enhanced OS benefit (HR, 0.75; 95% CI, 0.62 to 0.91; *p* = 0.0032) while it did not prolong PFS (HR, 0.84; 95% CI, 0.70 to 1.01) through introducing durvalumab combined with chemotherapeutics for ES-SCLC clinical cases in comparison to chemotherapy alone, though durvalumab plus tremelimumab within chemotherapeutics did not significantly improve OS (HR, 0.82; 95% CI, 0.68 to 1.00; *p* = 0.045) and PFS (HR 0.84, 95% CI 0.70 to 1.01) (10). The KEYNOTE-604 study illustrated that pembrolizumab plus chemotherapy significantly improved PFS (HR, 0.75; 95% CI, 0.61 to 0.91; *p* = 0.0023) and slightly prolonged OS (HR, 0.78; 95% CI, 0.63 to 0.97; *p* = 0.0164) compared with chemotherapy as initial therapy for ES-SCLC cases (11). The CA184-156 investigation revealed that ipilimumab plus chemotherapy failed to extend OS (HR, 0.94; 95% CI, 0.81 to 1.09; *p* = 0.3775) and slightly extend PFS (HR, 0.85; 95% CI, 0.75 to 0.97; *p* = 0.0161) versus chemotherapy alone within clinical cases having novel-diagnosed ES-SCLC (12). Founded upon such datasets, atezolizumab or durvalumab were approved by the US Food and Drug Administration (FDA) (13, 14) and the National Comprehensive Cancer Network (NCCN) for combination therapy with EP as a first-line option against ES-SCLC (15).

However, considering that there is no research to directly compare different immuno-chemotherapy regimens, it is not clear which therapeutic option must be recommended as initial treatment in such clinical cases. Based upon present healthcare scenarios and relevant stakeholders, we need more proof to validate different immuno-chemotherapy within oncology health care to provide effective medical leverage with decent costings. Consequently, this investigation employed recently reported randomized controlled trials (RCTs) for network meta-analysis (NMA) and cost-effectiveness analysis (CEA) for evaluating effectiveness, safety, and cost-effectiveness for immuno-chemotherapy and chemotherapy alone as the initial therapeutic option for ES-SCLC clinical cases, from a US payer perspective.

## 2. Methods

This work was guided by the PRISMA statement, which included a PRISMA NMA checklist and the consolidated health economic evaluation reporting standards statement (CHEERS) checklist (Supplementary Tables 2, 3 within the Supplementary material).

### 2.1. Search strategy and inclusion criteria

A systematic review and NMA were conducted for identifying eligible phase III RCTs to compare regimens containing ICIs plus chemotherapy in first-line treatment. We retrieved the Pubmed, Embase, Cochrane, and Web of Science databases for published articles written in English from Jan 1, 2000, to Nov 30, 2021, with the search terms “PD-1,” “PD-L1,” “immunotherapy,” “chemotherapy,” “extensive-stage small-cell lung cancer,” and “clinical trial” (Supplementary Table 1 in the Supplementary material). In addition, the investigation also focused on abstracts reported by the American Society of Clinical Oncology (ASCO) and the European Society of Medical Oncology (ESMO). Finally, relevant literature was manually screened to avoid missing articles.

Inclusion criteria: (1) patients diagnosed with ES-SCLC; (2) articles in which participants received both types of treatment, one of which was immuno-chemotherapy and the other was chemotherapy; (3) both treatment measures were in the initial treatment environment of ES-SCLC patients; (4) phase III RCTs; (5) the article had the most complete and updated data of the trial; (6) studies published in English. Studies not matching the inclusion criteria were excluded. YWZ and KL carried out literature retrieval and data extraction independently. Whenever duplicate studies were identified, the article having the most comprehensive and recent investigation data were included. Reviews / systematic reviews, meta-analyses, and CEAs were excluded from this investigation.

### 2.2. Data extraction and determination of bias risks

Details were extracted from identified articles, such as author, publication year, trial name or identification, treatment regimens of experimental groups and control groups, number of patients treated, HR of OS and PFS of the overall population, median OS and PFS, together with the incidence of grade 3/4 AEs from each included investigation. Additionally, the odds ratio (OR) of grade 3/4 AEs and the HR of OS and PFS of the population with BMs or non-brain metastases (NBMs) were extracted.

Individual RCT article bias risks were evaluated in line with the Cochrane Collaboration guideline (16), valuating multiple facets for RCT experimental designs, behavior, and detail descriptions. Seven tools were used to assess individual RCT results, namely: (1) random sequence generation, (2) allocation concealment, (3) blinding of participants and personnel, (4) blinding of outcome assessment, (5) incomplete outcome data, (6) selective reporting, and (7) other bias.

### 2.3. Statistical analysis

R software (version 4.1.1)^1^ with the package “netmeta” was employed for comparative analysis. We combined the HR and 95% CI that was collected. However, since just one RCT informed individual pair-wise comparisons, with paucity in datasets for evaluating heterogeneity across trials, a fixed-effect model was established. Consequently, the frequency method was employed for comparing effectiveness and safety for different schemes. The HR of OS and PFS, corresponding 95% CI, *p*-value, and OR of AEs were calculated. Subgroup analyses were performed on status with or without BMs. Finally, according to the obtained 95% Cl of HR and p-value, the best treatment schemes were sorted.

### 2.4. Cost-effectiveness analysis

#### 2.4.1. Model structure

A Markov model and decision tree having multiple health-parameters (PFS, progressive disease (PD), and death) (Supplementary Figure 5 in the Supplementary material) was established to assess costings and efficacy for different initial patient treatments for ES-SCLC. The Markov model cycle was determined to be 6 weeks based on the patient’s survival and dosing follow-up protocol. Since tremelimumab has not obtained obvious clinical benefits and was not listed, the decision trees included 5 initial therapeutic options: (1) atezolizumab plus chemotherapy, (2) durvalumab plus chemotherapy, (3) pembrolizumab plus chemotherapy, (4) ipilimumab plus chemotherapy, and (5) chemotherapy. Over time, the patient’s health status deteriorated and led to mortality, with more than 99% of the registered patients dead over the last 15 years. All patients started PFS status and could receive five kinds of initial treatment strategies randomly. Upon PD or unacceptable toxicity and AEs, some patients received topotecan as subsequent treatment, according to Koichi Goto’s recommendations (17); Other patients received supportive treatment (15). To better reflect the current clinical work, the study considered that patients received palliative treatment before the mortality event. All doses and dosing schedules for each treatment regimen were collected from corresponding RCTs (9–12) (Supplementary Table 5 in the Supplementary material).

The study adopted costings and influence from a 3% discounted rate per year (18). The outputs encompassed overall cost, life-years (LYs), quality-adjusted LYs (QALYs), and incremental cost-effectiveness ratios (ICERs). The study also focused on population CEA with or without BMs. Depending upon the U.S. consumer-price index, all costings related to healthcare services were inflated to the value of 2021, and willingness-to-pay (WTP) in the United States was $150,000 (19, 20). The Markov model used TreeAge Pro 2020® (TreeAge Software™, Williamstown, MA)^2^.

#### 2.4.2. Model survival and progression risk estimates

This research implemented GetData Graph Digitizer® (version 2.26)^3^ for gathering data from OS and PFS curve-strategy from RCTs. Consequently, we reconstructed the OS and PFS curves of chemotherapeutics patients depending upon Kaplan–Meier (KM) chemotherapeutic curves of four RCTs and such data were consequently employed for fitting parametric survival models. Peak-consistent Weibull distribution was chosen depending upon Akaike’s information criterion (AIC) and Bayesian information criterion (BIC) (Supplementary Table 6; Supplementary Figures 6, 7 in the Supplementary material) (21). Consequently, the study used Weibull distribution and obtained two-parameter, shape (γ) and scale (λ), which were determined through such a fit. This study employed Hoyle and Henley’s suggested methodology (22) (Table 1).

**Table 1:** Model parameters: baseline values, ranges, and distributions for sensitivity analysis.

Parameters	Baseline value	Range		References	Distribution
		Minimum	Maximum		
*Survival*					
Weibull survival model of OS of C	Scale = 0.010872,	–	–	(7–11)	–
	Shape = 1.750803				
Weibull survival model of PFS of C	Scale = 0.026945,	–	–		–
	Shape = 2.100966				
Weibull survival model of OS of AC	Scale = 0.01412,	–	–	(7, 8)	–
	Shape = 1.490903				
Weibull survival model of PFS of AC	Scale = 0.11144,		-		-
	Shape = 1.19819	–			
Weibull survival model of OS of DC	Scale = 0.022259,	–	–	(9)	–
	Shape = 1.334609				
Weibull survival model of PFS of DC	Scale = 0.15276,	–	–		-
	Shape = 0.92421				
Weibull survival model of OS of PC	Scale = 0.03787,	–	–	(10)	–
	Shape = 1.1735				
Weibull survival model of PFS of PC	Scale = 0.07424,	–	–		-
	Shape = 1.40271				
Weibull survival model of OS of IC	Scale = 0.008878,	–	–	(11)	–
	Shape = 1.790279				
Weibull survival model of PFS of IC	Scale = 0.02302	–	–		-
	Shape = 2.11942				
*Risk for main AEs in C group*					
Risk of neutropenia	0.29	0.23	0.35	(7–11)	Beta
Risk of anemia	0.12	0.10	0.15	(7–11)	Beta
Risk of thrombocytopenia	0.07	0.06	0.09	(7–11)	Beta
Risk of leucopenia	0.05	0.04	0.06	(7–11)	Beta
Risk of neutrophil count decreased	0.07	0.05	0.08	(7–11)	Beta
*Risk for main AEs in AC group*					
Risk of thrombocytopenia	0.10	0.08	0.12	(7)	Beta
Risk of neutropenia	0.23	0.18	0.27	(7)	Beta
Risk of anemia	0.14	0.11	0.17	(7)	Beta
Risk of neutrophil count decreased	0.14	0.11	0.17	(7)	Beta
Risk of leucopenia	0.05	0.04	0.06	(7)	Beta
*Risk for main AEs in DC group*					
Risk of neutropenia	0.24	0.19	0.29	(9)	Beta
Risk of anemia	0.09	0.07	0.11	(9)	Beta
Risk of thrombocytopenia	0.06	0.05	0.07	(9)	Beta
Risk of leucopenia	0.06	0.05	0.07	(9)	Beta
Risk of neutrophil count decreased	0.06	0.05	0.07	(9)	Beta
Risk of febrile neutropenia	0.06	0.05	0.07	(9)	Beta
Risk of hyponatraemia	0.06	0.05	0.07	(9)	Beta
*Risk for main AEs in PC group*					
Risk of neutropenia	0.44	0.35	0.52	(10)	Beta
Risk of anemia	0.16	0.13	0.19	(10)	Beta
Risk of thrombocytopenia	0.14	0.11	0.17	(10)	Beta
Risk of leucopenia	0.12	0.09	0.14	(10)	Beta
Risk of pneumonia	0.07	0.05	0.08	(10)	Beta
*Risk for main AEs in IC group*					
Risk of diarrhea	0.07	0.06	0.08	(11)	Beta
Risk of anemia	0.08	0.06	0.10	(11)	Beta
Risk of neutropenia	0.14	0.11	0.17	(11)	Beta
Risk of neutrophil count decreased	0.07	0.06	0.08	(11)	Beta
Utility					
Utility PFS in first-line treatment	0.673	0.54	0.81	(23)	Beta
Utility PD	0.473	0.38	0.57	(23, 24)	Beta
*Disutility due to AEs*					
Neutropenia	0.09	0.07	0.11	(24)	Beta
Anemia	0.073	0.06	0.09	(24)	Beta
Leucopenia	0.09	0.07	0.11	(24)	Beta
Pneumonia	0.09	0.07	0.11	(25)	Beta
Thrombocytopenia	0.65	0.52	0.78	(24)	Beta
Neutrophil count decreased	0.09	0.07	0.11	(24)	Beta
Febrile Neutropenia	0.09	0.07	0.11	(24)	Beta
Hyponatraemia	0.094	0.08	0.11	(23)	Beta
Diarrhea	0.22	0.18	0.26	(23)	Beta
AEs disutility for AC	0.09	0.07	0.11	(23)	Beta
AEs disutility for DC	0.094	0.08	0.11	(23)	Beta
*Drug cost, $/per cycle*					
Atezolizumab	19,140	15,312	22,968	(26)	Gamma
Durvaluma	23,059	18,447	27,671	(26)	Gamma
Pembrolizumab	21,102	16,881	25,322	(26)	Gamma
Ipilimumab	222,107	177,686	266,539	(26)	Gamma
Etoposide	88	70	105	(26)	Gamma
Carboplatin	52	41	62	(26)	Gamma
Topotecan	141	113	169	(26)	Gamma
*Cost of AEs, $*					
Chemotherapy	15,168	12,134	18,202	(20, 23, 25, 27)	Gamma
Atezolizumab plus chemotherapy	15,866	12,693	19,039	(20, 23, 25, 27)	Gamma
Durvaluma plus chemotherapy	15,499	12,399	18,599	(20, 23, 25, 27)	Gamma
Pembrolizumab plus chemotherapy	20,581	16,465	24,697	(20, 23, 25, 27)	Gamma
Ipilimumab plus chemotherapy	8,536	6,829	10,243	(20, 23, 25, 27)	Gamma
Laboratory per cycle	315	252	378	(22)	Gamma
Tumor imaging per cycle	231	185	277	(24)	Gamma
Administration per cycle	140	112	168	(24)	Gamma
Best supportive care per cycle	3,299	2,639	3,959	(27)	Gamma
Death associated costs per patient	9,433	7,546	11,320	(23)	Gamma
Discount rate	0.03	–	–	(17)	–

OS, overall survival; PFS, progression-free survival; C; chemotherapy; AC; atezolizumab plus chemotherapy; DC; durvaluma plus chemotherapy; PC; pembrolizumab plus chemotherapy; IC; ipilimumab plus chemotherapy; AEs, adverse events.

Time-dependency transition probabilities(tp) are vital for such modeling evaluations. Tp for individual Markov cycles was determined depending upon following formula: $\text{tp}\left({\text{t}}_{\text{u}}\right)=1-\exp\left\{\text{λ}{\left(\text{t}-\text{u}\right)}^{\text{γ}}-\lambda {\text{t}}^{\text{γ}}\right\}\;\left(\lambda >0\text{,}\gamma >0\right)$ (26).

where Markov cycle = u, arrival at state t after u Markov cycles i = tu, respectively.

#### 2.4.3. Cost and utility estimates

This study considered just immediate medical expenses from a US payer perspective, including drug costs (24), AEs costs (with the assumption that AEs occurred within just 1 cycle during PFS and PD states) (20, 23, 25, 27), administration, tumor imaging, laboratory (23), and death associated costs (25), and best supportive care (28).

Based on four RCTs and clinical practice, carboplatin was selected as the main treatment regimen in the chemotherapeutics group. Once drug cost per cycle was determined, assuming the patient was male-gender, 65 years old, weighing 70 Kg, the height of 170, and body-surface-area 1.84m^2^, area-under-concentration (AUC) curve of 5 mg/ml/min, together with presumed serum creatinine being 1 (29). Medical monitoring costings encompassed financial charges for computed tomography or magnetic resonance imaging (at six-week intervals for the initial 48 weeks and 9-week intervals afterward) (9, 11). This study solely added costings for managing grade 3/4 AEs (frequency > 5%) within this model that had distinctly varying probabilities across RCT arms. The entirety of costings linked to healthcare provisions was inflated to correspondent values in 2021, depending upon the US consumer-price index (Table 1).

We used previously published utilities of 0.673 and 0.473 (25) as the mean health utility value for PFS and PD states, accordingly. This investigation also included dis-utility values of grade 3/4 AEs within analysis (23, 25, 27).

#### 2.4.4. Sensitivity and scenario analysis

This investigation employed serial sensitivity evaluating predictions for modeling outcome uncertainties. One-way sensitivity evaluation was performed within a variance of 20% baseline values, depending upon varying values for a specific parameter (within the expected range) and pre-determined methodologies for examining individual parameter-driven influences over ICERs (23). This investigation additionally conducted probabilistic sensitivity analyses for evaluating the probability of efficacy by therapeutic regimens through 10,000 Monte Carlo repetitions. A cost-effectiveness adequacy curve for individual therapeutic modalities was assessed to present probabilities of cost-effectiveness.

Subgroup analyses were performed on status with or without BMs of four RCTs. Due to insufficient data for several RCTs, this investigation used identical pooled chemotherapeutics KM to obtain depending upon subgroup-defined HRs, as described by Hoyle (30) for lack of OS and PFS curves regarding BMs status of subgroups.

In addition, we conducted a scenario analysis, where ICIs maintenance phase until death after 4 cycles of first-line treatment, for evaluating if maintenance time for ICIs had a major influence on this investigation’s outcomes.

## 3. Results

### 3.1. Included studies

We searched 200 records, and 63 eligible articles were searched in full text. After screening, four cluster RCTs, involving 2,793 patients, were included (Supplementary Table 4; Supplementary Figure 1 in the Supplementary material). These patients received first-line treatment with atezolizumab plus chemotherapy (*n* = 201 patients), durvalumab plus chemotherapy (*n* = 268 patients), durvalumab with tremelimumab plus chemotherapy (*n* = 268 patients), pembrolizumab plus chemotherapy (n = 228 patients), ipilimumab plus chemotherapy (*n* = 478 patients), and chemotherapy (n = 1,172 patients).

### 3.2. Risk-bias proof evaluations

We employed RevMan® (version 5.4) to summarize risk-bias (Supplementary Figure 2 in the Supplementary material). Two studies were designated as cluster RCTs and employed randomization concealment. Three investigations were described as double-blinded. Three investigations were found to have reduced risk-bias due to blinding of outcome evaluation, while all studies were judged to have a low risk of bias for incomplete outcome data and selective reporting.

### 3.3. Results of the network meta-analysis

The network plots were built using R software (version 4.1.1), including five immuno-chemotherapy regimens (atezolizumab plus chemotherapy, durvalumab plus chemotherapy, durvalumab with tremelimumab plus chemotherapy, pembrolizumab plus chemotherapy, and ipilimumab plus chemotherapy) and one control regimen (chemotherapy) (Supplementary Figure 3 in the Supplementary material). Indirect comparison showed that atezolizumab plus chemotherapy (HR, 0.76; 95% CI, 0.60 to 0.96 and HR, 1.32; 95% CI, 1.05 to 1.66), durvalumab plus chemotherapy (HR, 0.75; 95% CI, 0.62 to 0.91 and HR, 1.33; 95% CI, 0.62 to 0.91), durvalumab with tremelimumab plus chemotherapy (HR, 0.82; 95% CI, 0.68 to 0.99 and HR, 1.23; 95% CI, 1.01 to 1.48), and pembrolizumab plus chemotherapy (HR, 0.80; 95% CI, 0.65 to 0.99 and HR, 1.25; 95% CI, 1.01 to 1.55) had significant statistical improvement compared with chemotherapy in OS, and atezolizumab plus chemotherapy (HR, 0.77; 95% CI, 0.63 to 0.95 and HR, 1.30; 95% CI, 1.06 to 1.60), durvalumab plus chemotherapy (HR, 0.80; 95% CI, 0.66 to 0.97 and HR, 1.25; 95% CI, 0.66 to 0.97), pembrolizumab plus chemotherapy (HR, 0.75; 95% CI, 0.61 to 0.92 and HR, 1.33; 95% CI, 0.61 to 0.92), and ipilimumab plus chemotherapy (HR, 0.85; 95% CI, 0.75 to 0.97 and HR, 1.18; 95% CI, 0.75 to 0.97) had significant statistical improvement compared with chemotherapy in PFS in the overall population. No statistically significant differences in PFS and OS were found between the five immuno-chemotherapy regimens. In the population with BMs, durvalumab plus chemotherapy (HR, 0.76; 95% CI, 0.62 to 0.93 and HR, 1.32; 95% CI, 1.08 to 1.60) and ipilimumab plus chemotherapy (HR, 0.63; 95% CI, 0.41 to 0.98 and HR, 1.58; 95% CI, 1.02 to 2.44) were significantly improved in OS and PFS in comparison to chemotherapy. In the population with NBMs, atezolizumab plus chemotherapy (HR, 0.74; 95% CI, 0.58 to 0.94 and HR, 1.35; 95% CI, 1.06 to 1.72), durvalumab with tremelimumab plus chemotherapy (HR, 0.81; 95% CI, 0.67 to 0.99 and HR, 1.24; 95% CI, 1.06 to 1.72), pembrolizumab plus chemotherapy (HR, 0.75; 95% CI, 0.60 to 0.94 and HR, 1.33; 95% CI, 1.07 to 1.67); atezolizumab plus chemotherapy (HR, 0.75; 95% CI, 0.60 to 0.93 and HR, 1.33; 95% CI, 1.07 to 1.66), durvalumab plus chemotherapy (HR, 0.80; 95% CI, 0.66 to 0.97 and HR, 1.25; 95% CI, 1.04 to 1.51), pembrolizumab plus chemotherapy (HR, 0.69; 95% CI, 1.04 to 1.51 and HR, 1.45; 95% CI, 1.17 to 1.80), and ipilimumab plus chemotherapy (HR, 0.85; 95% CI, 0.78 to 0.97 and HR, 1.18; 95% CI, 1.04 to 1.34) were significantly improved in PFS compared with chemotherapy.

**Figure 1:**
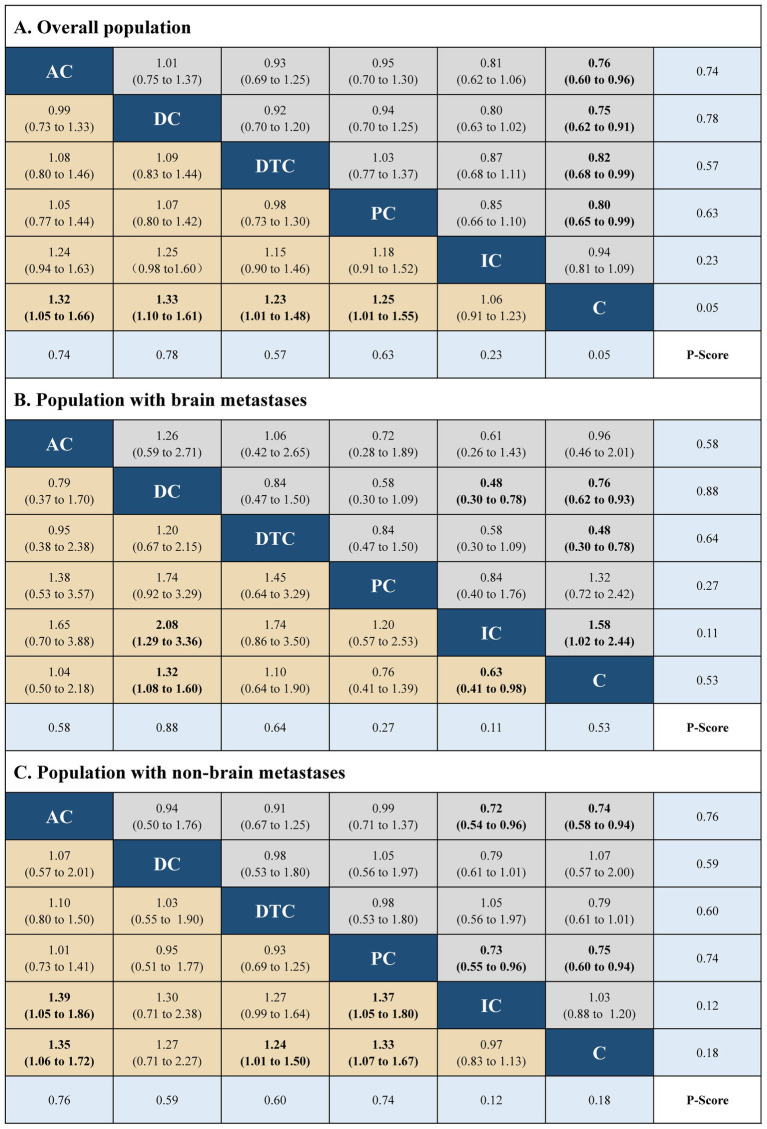
Hazard ratios (gray and brown cell) and *p*-values (blue cell) of the network meta-analysis of the overall survival in the overall population **(A)**, population with brain metastases **(B)**, and population with non-brain metastases **(C)**. AC, atezolizumab plus chemotherapy; DC, durvaluma plus chemotherapy; DTC, durvalumab with tremelimumab plus chemotherapy; PC, pembrolizumab plus chemotherapy; IC, ipilimumab plus chemotherapy; C, chemotherapy.

**Figure 2:**
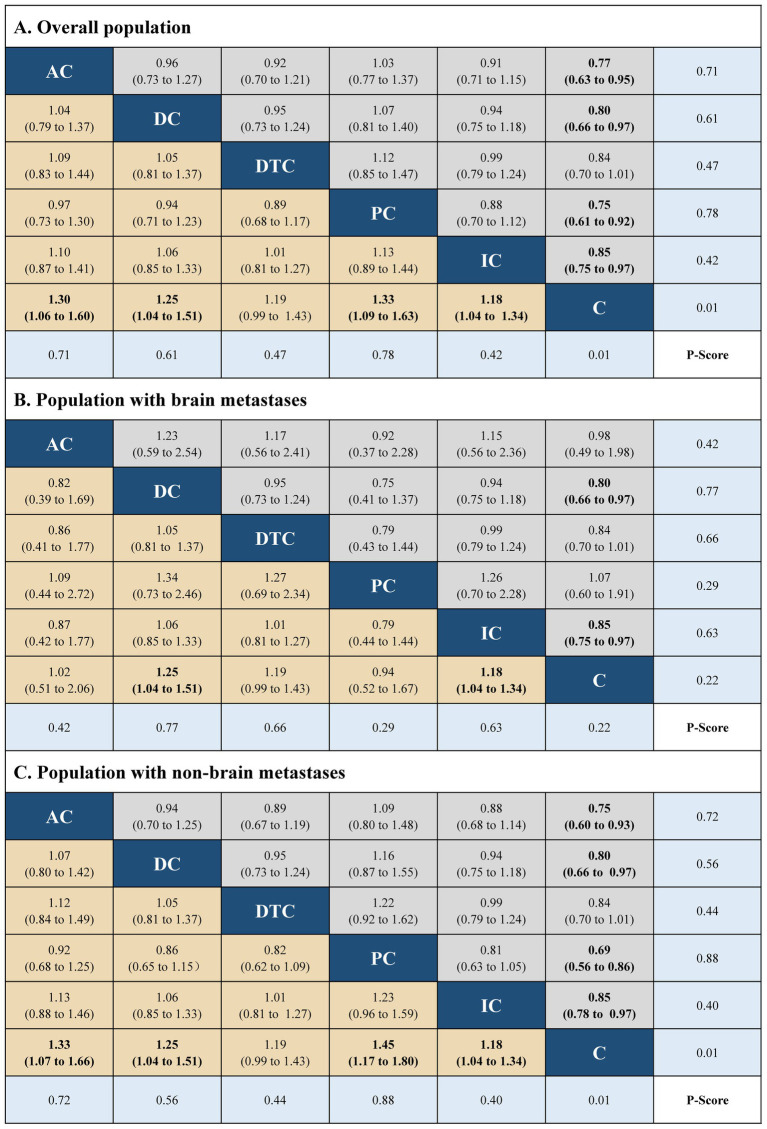
Hazard ratios (gray and brown cell) and p-values (blue cell) of the network meta-analysis of the progression-free survival in the overall population **(A)**, population with brain metastases **(B)**, and population with non-brain metastases **(C)**. AC, atezolizumab plus chemotherapy; DC, durvaluma plus FIGURE 2 (Continued)chemotherapy; DTC, durvalumab with tremelimumab plus chemotherapy; PC, pembrolizumab plus chemotherapy; IC, ipilimumab plus chemotherapy; C, chemotherapy.

The best treatment results were ranked according to *value of p* (individual outcomes), where raised values were more successful. Among the overall populations, the regimen having peak *value of p* for OS was durvalumab plus chemotherapy (*p* = 0.78), followed by atezolizumab plus chemotherapy (*p* = 0.74). However, the regimen with the highest *value of p* for PFS was pembrolizumab plus chemotherapy (*p* = 0.78), followed by atezolizumab plus chemotherapy (*p* = 0.71), durvalumab plus chemotherapy (*p* = 0.61). The regimens with the highest *value of p* for OS and PFS in the population with NBMs were durvalumab plus chemotherapy (*p* = 0.88 and *p* = 0.77). Among the population with BMs, the regimen with the highest *value of p* for OS and PFS were atezolizumab plus chemotherapy (*p* = 0.76) and pembrolizumab plus chemotherapy (*p* = 0.88), respectively. The results of indirect comparisons and the *p*-values of the PFS and OS of each regimen were shown in Figures 1, 2, respectively.

The safety table and forest plot showed that the five immuno-chemotherapy schemes have considerable safety profiles for any grade AEs (Supplementary Figure 4; Supplementary Table 7 in the Supplementary material). The general safety of immuno-chemotherapy ranked from high to low for all AEs was as follows: chemotherapy (probability 90%), ipilimumab plus chemotherapy (56%), atezolizumab plus chemotherapy (52%), durvalumab plus chemotherapy (52%), pembrolizumab plus chemotherapy (37%), and durvalumab with tremelimumab plus chemotherapy (13%). The general safety of immuno-chemotherapy ranked from high to low for severe AEs was as follows: chemotherapy (70%), atezolizumab plus chemotherapy (63%), durvalumab plus chemotherapy (57%), pembrolizumab plus chemotherapy (43%), ipilimumab plus chemotherapy (38%), and durvalumab with tremelimumab plus chemotherapy (31%).

### 3.4. Results of the cost-effectiveness analyses

Regarding ES-SCLC cases, this investigation expressed the output effects of five interventions by QALYs (LYs), from more to less was as follows: atezolizumab plus chemotherapy (1.02 QALYs and 1.91 LYs), durvalumab plus chemotherapy (1.01 QALYs and 1.90 LYs), pembrolizumab plus chemotherapy (0.93 QALYs and 1.80 LYs), ipilimumab plus chemotherapy (0.85 QALYs and 1.55 LYs), and chemotherapy (0.77 QALYs and 1.44 LYs). The least total cost of each treatment regimen was ranked from high to low as follows: the total cost of ipilimumab plus chemotherapy was the highest, which was $568,657, followed by pembrolizumab plus chemotherapy ($241,682), durvalumab plus chemotherapy ($229,620), and atezolizumab plus chemotherapy ($213,988). The lowest total cost of chemotherapy was $133,625. Post-further analysis, atezolizumab plus chemotherapy, durvalumab plus chemotherapy, pembrolizumab plus chemotherapy, ipilimumab plus chemotherapy obtained an ICER of $321,452/QALY, $399,978/QALY, $675,358/QALY, and $5,437,894/QALY, respectively, compared with chemotherapy. The baseline results and pairwise comparison of ICER were shown in Table 2; Supplementary Table 8.

**Table 2:** Baseline results.

Treatment	Total cost $	LYs	ICER $/LY ^a^	QALYs	ICER $/QALY^b^
*Overall population*					
Chemotherapy	133,625	1.44	NA	0.77	NA
Atezolizumab plus Chemotherapy	213,988	1.91	170,985	1.02	321,452
Durvaluma plus Chemotherapy	229,620	1.90	208,685	1.01	399,978
Pembrolizumab plus Chemotherapy	241,682	1.80	300,158	0.93	675,358
Ipilimumab plus Chemotherapy	568,657	1.55	3,954,836	0.85	5,437,894
*Population with brain metastases*					
Chemotherapy	133,625	1.44	NA	0.77	NA
Atezolizumab plus Chemotherapy	181,487	1.49	957,240	0.81	1,196,550
Durvaluma plus Chemotherapy	208,187	1.67	324,183	0.89	621,350
Pembrolizumab plus Chemotherapy	191,643	1.19	Dominated^c^	0.64	Dominated^c^
Ipilimumab plus Chemotherapy	517,993	1.15	Dominated^c^	0.65	Dominated^c^
*Population with non-brain metastases*					
Chemotherapy	133,625	1.44	NA	0.77	NA
Atezolizumab plus Chemotherapy	202,362	1.71	254,582	0.93	429,606
Durvaluma plus Chemotherapy	205,489	1.64	359,320	0.87	718,640
Pembrolizumab plus Chemotherapy	235,428	1.63	535,805	0.85	1,272,538
Ipilimumab plus Chemotherapy	540,073	1.45	40,644,800	0.79	20,322,400

a Compared to Chemotherapy ($/LY).

b Compared to Chemotherapy ($/QALY).

c Treatment showed lower effectiveness and higher cost, as compared with the chemotherapy.

ICER, incremental cost-effectiveness ratio; LY, life-year; QALY, quality-adjusted life-year.

The one-way sensitivity analysis showed it was highly sensitive for the utility of PD against chemotherapy. Other considerable influences were the risk of neutropenia in the chemotherapy group or immuno-chemotherapy group, cost of ICIs, and utility of PD. Alternative factors encompassed within sensitivity analysis, such as the costing and disutilities of AEs, had a minimal impact on ICER (Supplementary Figure 8 in the Supplementary material).

**Figure 3:**
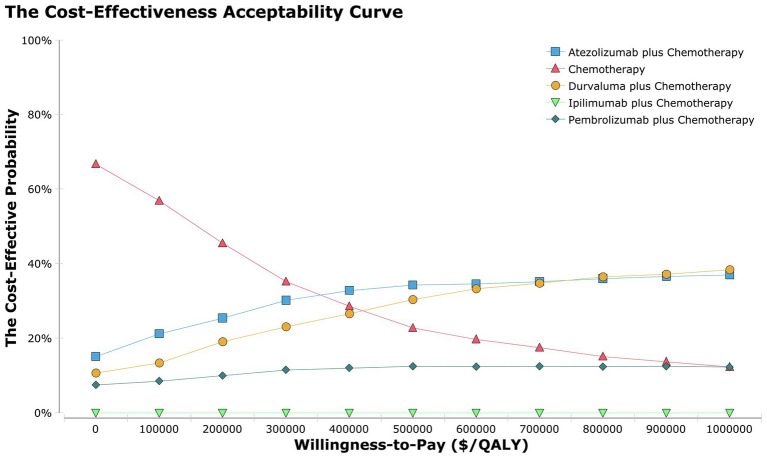
The cost-effectiveness acceptability curves for the atezolizumab plus chemotherapy, durvaluma plus chemotherapy, pembrolizumab plus chemotherapy, ipilimumab plus chemotherapy strategies compared to the chemotherapy strategy in the overall population.

Dataset outcomes for acceptability curves (Figure 3) and ICER scatterplot (Supplementary Figure 9 in the Supplementary material) demonstrated that the probability of atezolizumab plus chemotherapy, durvalumab plus chemotherapy, pembrolizumab plus chemotherapy, and ipilimumab plus chemotherapy being cost-effective were 32, 29 10, 0% in the overall population, respectively, compared with that of chemotherapy a WTP threshold of $150,000.

Regarding patient-populations experiencing BMs and NBMs, ICERs for atezolizumab plus chemotherapy, durvalumab plus chemotherapy, pembrolizumab plus chemotherapy, ipilimumab plus chemotherapy versus chemotherapy were $5,437,894 and $429,606, $621,350 and $718,640, $-446,292, and $1,272,538, and $-3,203,067 and $20,322,400 per QALY, respectively (Table 2). Results of ICER scatterplot (Supplementary Figures 10, 11 in the Supplementary material) showed that the probability of atezolizumab plus chemotherapy, durvalumab plus chemotherapy, pembrolizumab plus chemotherapy, and ipilimumab plus chemotherapy being cost-effective were 12 and 34%, 39 and 20%, 0 and 19%, 0 and 0% in the population with BMs and NBMs, compared with that of chemotherapy a WTP threshold of $150,000, respectively.

Scenario-analysis outcomes suggested that ICIs maintenance therapy resulted in the health costings linked to initial treatment increasing drastically, though this investigation’s outcome was not altered. This investigation assumed that clinical cases had ICIs maintenance therapy until death after 4 cycles of initial treatment, whereby health costs of the first-line atezolizumab plus chemotherapy, durvalumab plus chemotherapy, pembrolizumab plus chemotherapy, and ipilimumab plus chemotherapy were $279,513, $326,911, $306,097, and $1,271,747, respectively. An the ICERs were $355,700, $519,417, $731,140, and $5,963,788 per QALY, respectively.

## 4. Discussion

Recently, the promotion of ICIs has vastly shifted therapeutic options for ES-SCLC patients. Some encouraging results of phase III clinical studies demonstrated that introducing atezolizumab, durvalumab, durvalumab plus tremelimumab, pembrolizumab, and ipilimumab to chemotherapy shows clinical activity. Considering that these expensive drugs have brought a heavy burden on social health resources and patients, it is unclear which treatment regimen has the best efficacy and safety in the first-line treatment of ES-SCLC. Consequently, this investigation pioneered a comprehensive comparative clinical trial of immuno-chemotherapy and proved that one of the ICIs has better efficacy, safety, and overall economic outcomes. The results of NMAs indicated that atezolizumab plus chemotherapy and durvalumab plus chemotherapy regimens produced more survival benefits in patients with NBMs and BMs than other immuno-chemotherapy regimens and chemotherapy, respectively. Furthermore, the survival advantages of atezolizumab plus chemotherapy and durvalumab plus chemotherapy translated into the highest QALYs in patients with NBMs and BMs, respectively. All five immuno-chemotherapy regimens were associated with all levels of AEs risk, and ipilimumab plus chemotherapy strategy was linked to lowered risk for all-grade AEs (all levels) in comparison to chemotherapy. Unexpectedly, the safety of immuno-chemotherapy regimens is lower than that of chemotherapy strategy, which could be due to the combined regimens summarize AEs of ICIs and chemotherapy. Consequently, this assessment reflects the universal profiles of the current research results.

The baseline results of the CEA indicated that atezolizumab plus chemotherapy and durvalumab plus chemotherapy were the most effective strategies and provided the best treatment outcome in the NBMs and BMs populations, respectively. When it talks about cost-effectiveness according to relevant studies, immuno-chemotherapy regimens would be favored by clinical cases having reduced HRs for OS, while in patients with higher HRs it can become worse than chemotherapy (25, 31). Although atezolizumab plus chemotherapy and durvalumab plus chemotherapy provided 1.02 and 0.89 QALYs in patients with NBMs and BMs, respectively, whose QALYs were much higher than the other four treatment measures, they increased the survival benefit by 0.25 and 0.12 QALYs and the additional cost of $80,363 and $74,562, resulting in an ICER = 321,452 and 621,350/QALY, that is higher than WTP in the US, making it not cost-effective, in comparison to chemotherapy, respectively. Finally, modeling outcomes demonstrated that neither treatment plans were cost-effective in comparison to chemotherapy, in line with outcomes of several past investigations. However, chemotherapy alone was not enough to greatly improve the survival and prognosis of patients with ES-SCLC. Therefore, in addition to chemotherapy in first-line treatments, the most effective treatment strategy was to use atezolizumab plus chemotherapy for NBM cases and durvalumab plus chemotherapy for BM cases. Sensitivity analysis shows that the utility of PD was the most important factor influencing ICER value, followed by the incidence of AEs, and the price of ICIs are also factors that cannot be ignored. Since the price of ICIs is much higher than chemotherapy in the US, subsequent probabilistic sensitivity analysis results confirmed that atezolizumab plus chemotherapy and durvalumab plus chemotherapy were cost-effective in 32, 29, and 12%, 39% of the overall population and population with BMs, respectively. The results of the acceptable curve revealed that the US-based ICER value was affected by the shift in WTP value, while the US-based WTP value was affected by the *per capita* GDP. The average *per capita* US-based GDP value was adopted in our investigation (32). However, the *per capita* GDP of different regions in the US varies, so for several economically underdeveloped regions, the optimal strategy could be chemotherapy among the overall population. Regarding economically developed regions, atezolizumab plus chemotherapy and durvalumab plus chemotherapy were the preferred treatment options for the overall population and brain metastases, respectively.

The current assessment has several implications. On the one hand, patient survival has improved significantly with the introduction of ICIs. However, data was scarce for its efficacy within BM cases, and few clinical trials have been conducted for BMs alone. Patients with BMs were either excluded or only included in subgroups within key trials. The brain micro-environment itself has immunosuppressive effects, so it can promote the development of various tumor tissues and block anti-tumor immune responses (33–35). It is currently well established that chemotherapy can increase the efficacy of ICIs (36). Therefore, combination strategies may be more appropriate. For ES-SCLC, only the CASPIAN trial among our included studies demonstrated a trend of OS benefit in a small subgroup of patients with baseline BMs (55/805, 7%). HR for OS was 0.79 (95% CI, 0 44 to 1.41) (10). In non-small cell lung cancer (NSCLC), Powell et al. conducted a meta-analysis for three trials KEYNOTE-189,021, and 407, including baseline BMs (171/1298, 13%), and concluded that HR for OS was 0.48 (95% CI, 0.32 to 0.70) in the baseline BMs group treated with immuno-chemotherapy. In melanoma, the NIBIT trial included asymptomatic BM patients (n = 20/86, 36%) with a median OS of 12.7 months (95% CI, 2.7 to 22.7) (37). It should be noted that, from the perspective of patients with BMs and ES-SCLC, the high price of anti-cancer drugs can make cancer patients face huge financial toxicity (38). Regarding the balance of the health care system, ensuring that patients with specific characteristics have access to safe, effective, and innovative treatments is as important as minimizing economic toxicity.

On the other hand, immunotherapy was improving the therapeutic efficacy of SCLC. Physicians and administrators need to select proper patients who can benefit from this type of therapy to maintain our healthcare system and establishing prognostic and response predictive markers was critical. PD-L1 expression, tumor mutational burden (TMB), and tumor-infiltrating lymphocytes (TILs) can be reliable prognostic biomarkers in small-cell lung cancer (SCLC) (39–41). However, our study did not perform an analysis of biomarkers, so further studies are needed in future work to explore biomarkers to determine which patients with heterogeneous diseases are likely to benefit more from treatment so that treatment can be tailored to the individual.

Although this study has important strengths, some limitations should be considered. Firstly, when using the NMA method to indirectly compare immuno-chemotherapy regimens, we assumed that the included studies did not differ in patient characteristics and summarized the chemotherapy groups. Secondly, the inference of long-term survival benefit is depending upon short-term survival data of each experiment, which will alter upon change of long-term follow-up. This is an inevitable limitation in our model. Consequently, it is necessary to evaluate the concordance of such modeled health outcomes with real-world data. Thirdly, for enhanced analysis, this investigation assumed that all chemotherapy regimens used carboplatin, which was safer in the clinic. The cost of carboplatin was higher than that of cisplatin, so the cost of chemotherapy can be overestimated. However, sensitivity analysis demonstrated that the cost of carboplatin has little impact on the model results. Fourthly, several trials lacked survival data from subgroups, and the original group balance was produced by Hoyle’s methods. Consequently, the results of the subgroups analysis should be interpreted carefully. Fifth, this investigation analyzed the cost-effectiveness of patients with or without BMs. However we did not investigate the economic results of other subgroups, such as age, gender, smoking status, and liver metastasis. Sixth, due to the lack of complete QoL data to calculate the utility values, we referred the mean health utility value of NSCLC in PD state, and corrected the utility values by considering the disutility values of AEs and only 3/4 AEs were included, which might lead to overestimates or underestimates of the utility values. Finally, this investigation did not include social costs, including those related to the informal and non-health sectors.

In conclusion, immuno-chemotherapy regimens appear to be superior to standard chemotherapy. Among the five immuno-chemotherapy strategies, atezolizumab plus chemotherapy regimen seem to have the best effect on ES-SCLC patients other than BMs; durvalumab plus chemotherapy option can be a favorable condition for the population with BMs. Whereby, from the perspective of the US payer, the first-line use of four clinically effective immuno-chemotherapy regimens to treat ES-SCLC patients is not cost-effective in comparison to chemotherapy, though atezolizumab plus chemotherapy regimen can provide a more effective balance across ICER and QALYs in the overall population. Within BM clinical cases, durvalumab plus chemotherapy program obtain more health benefits. This finding can help physicians make decisions in clinical work and aid policy formulation in medical reimbursement.

## Data availability statement

The original contributions presented in the study are included in the article/Supplementary material, further inquiries can be directed to the corresponding author.

## Ethics statement

This article is based on previously conducted studies and does not contain any new studies with human participants or animals performed by any of the authors, it does not require the approval of the independent ethics committee.

## Author contributions

YZ, KL, QY, MZ, and LP: designed experiment, analyzed the data, wrote the manuscript, and complete the revision. YZ and KL: performed the experiments. LP and MZ: contributed analysis tools and funding. All authors have read and approved the manuscript.

## Conflict of interest

The authors declare that the research was conducted in the absence of any commercial or financial relationships that could be construed as a potential conflict of interest.

## Publisher’s note

All claims expressed in this article are solely those of the authors and do not necessarily represent those of their affiliated organizations, or those of the publisher, the editors and the reviewers. Any product that may be evaluated in this article, or claim that may be made by its manufacturer, is not guaranteed or endorsed by the publisher.

## Footnotes

^1^ http://www.r-project.org

^2^ https://www.treeage.com

^3^ http://www.getdata-graph-digitizer.com/index.php
