# Supplementary material for: First-line Immuno-chemotherapy for extensive-stage small-cell lung cancer: A network meta-analysis and cost-effectiveness analysis
Source: Front Public Health. 2023 Mar 16;11:1028202. doi: 10.3389/fpubh.2023.1028202 (PMC10061061; doi:10.3389/fpubh.2023.1028202)
Supplement: Supplementary file 1 [file Data_Sheet_1.PDF]

eFigure 1. Flow diagram of the study selection process.
eFigure 2. Risk of bias of evidence assessment.
eFigure 3. Network plots.
eFigure 4. The forest plots of AEs.
eFigure 5. Model structure for cost-effectiveness analysis.
eFigure 6. OS Kaplan-Meier Curve Fitting and Extrapolation.
eFigure 7. PFS Kaplan-Meier Curve Fitting and Extrapolation.
eFigure 8. The one-way sensitivity analyses.
eFigure 9. Probability Sensitivity Analysis Scatter Plot of the overall populations.
eFigure 10. Probability Sensitivity Analysis Scatter Plot of the population with brain metastases.
eFigure 11. Probability Sensitivity Analysis Scatter Plot of the population with non-brain metastases.
eTable 1. Search strategy.
eTable 2. PRISMA NMA Checklist.
eTable 3. CHEERS Checklist.
eTable 4. List of the studies included in the meta-analysis.
eTable 5. Drug dose and cost.
eTable 6. Summary of statistical goodness-of-fit of K-M curve.
eTable 7. Safety profiles.
eTable 8. Pairwise comparison of ICER ($/QALY).

eFigure 1. Flow diagram of the study selection process.

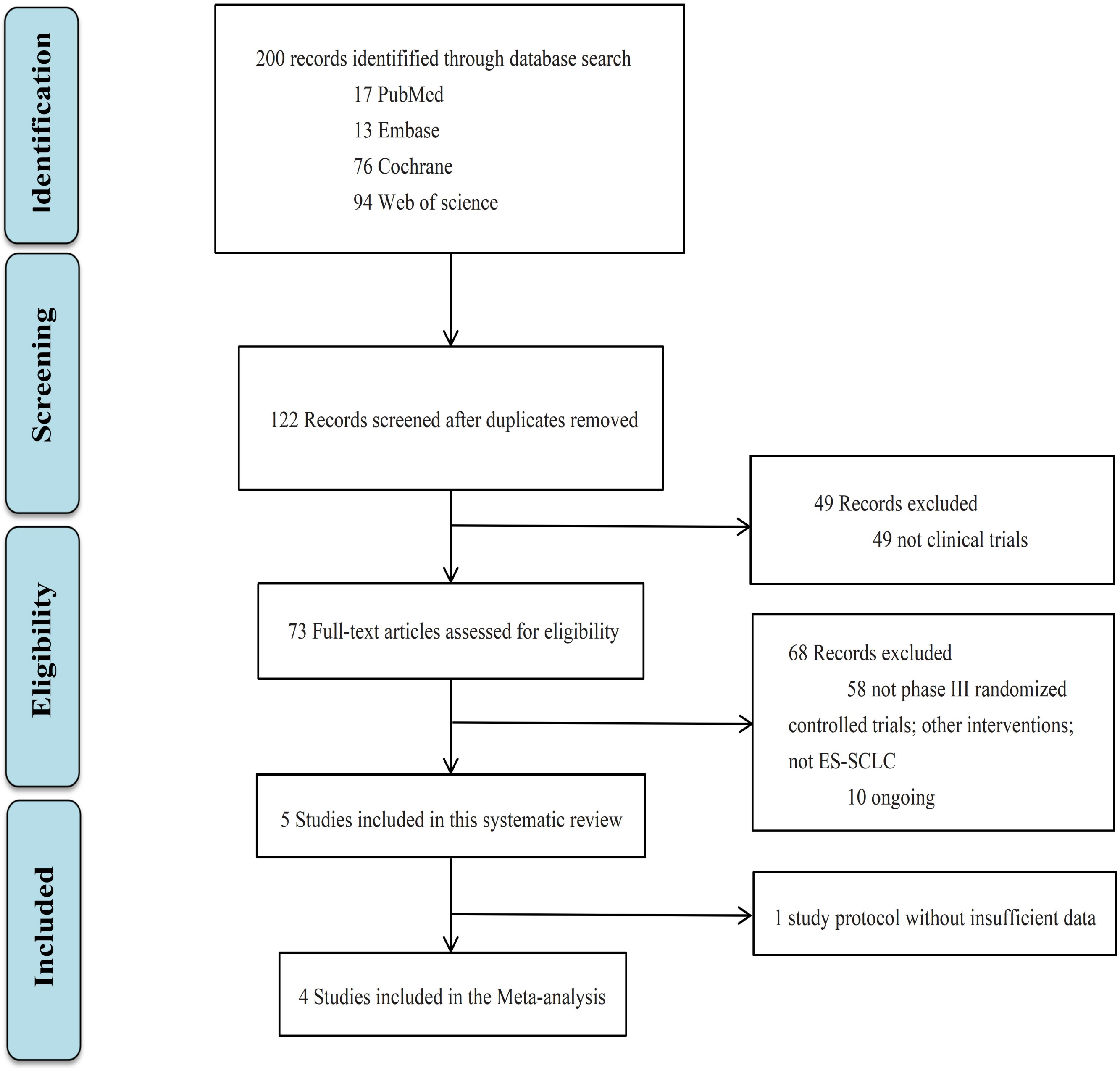

eFigure 2. Risk of bias of evidence assessment.

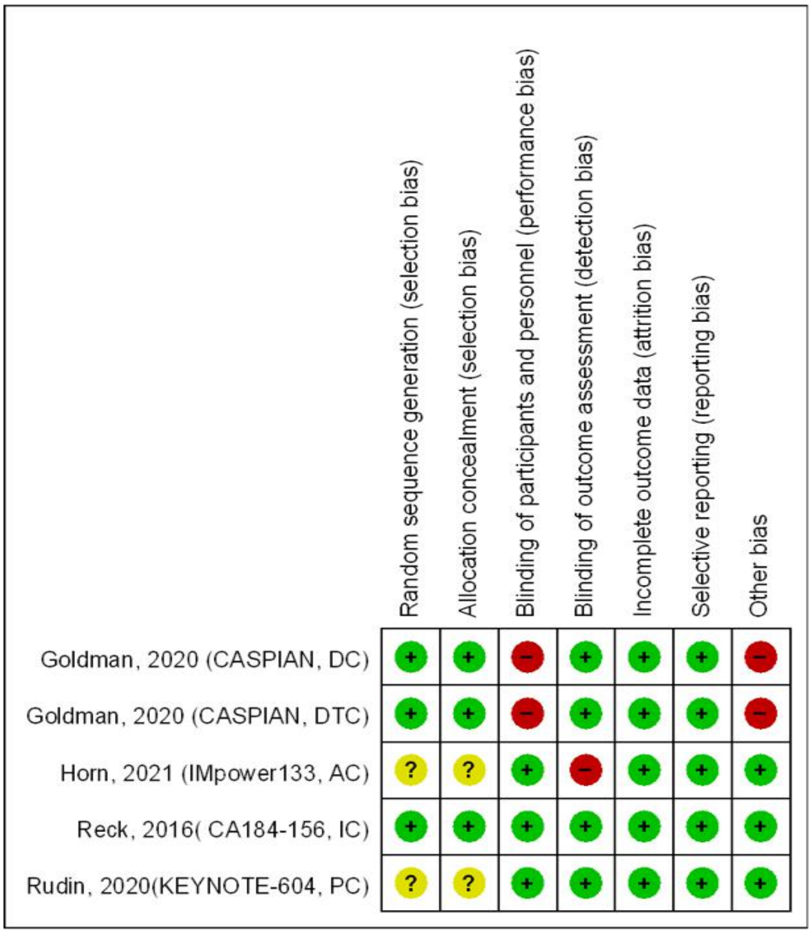

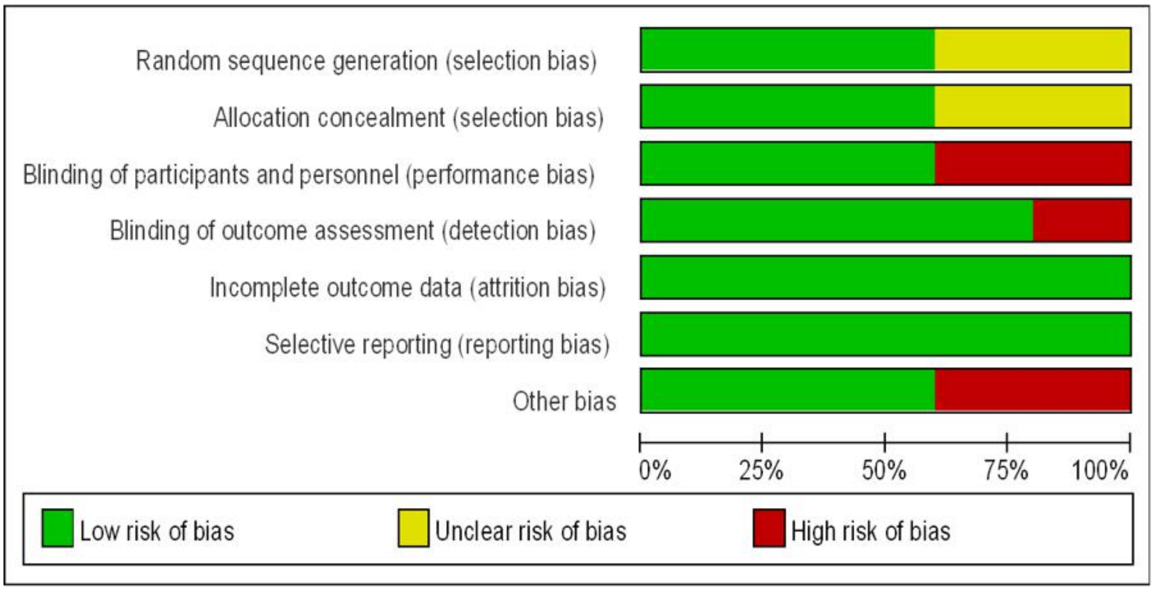

eFigure 3. Network plots.

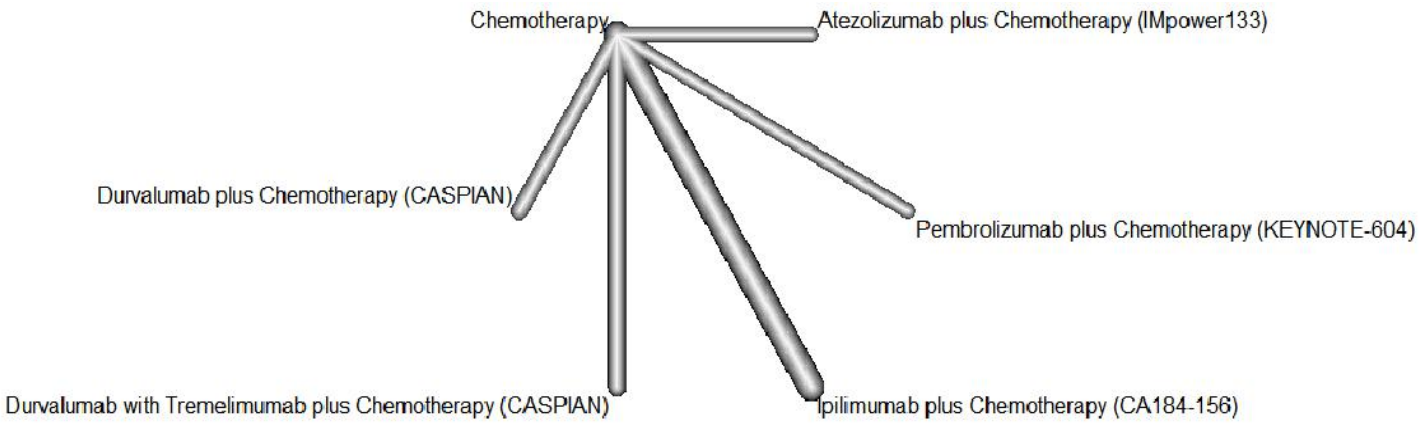

eFigure 4. The forest plots of AEs.

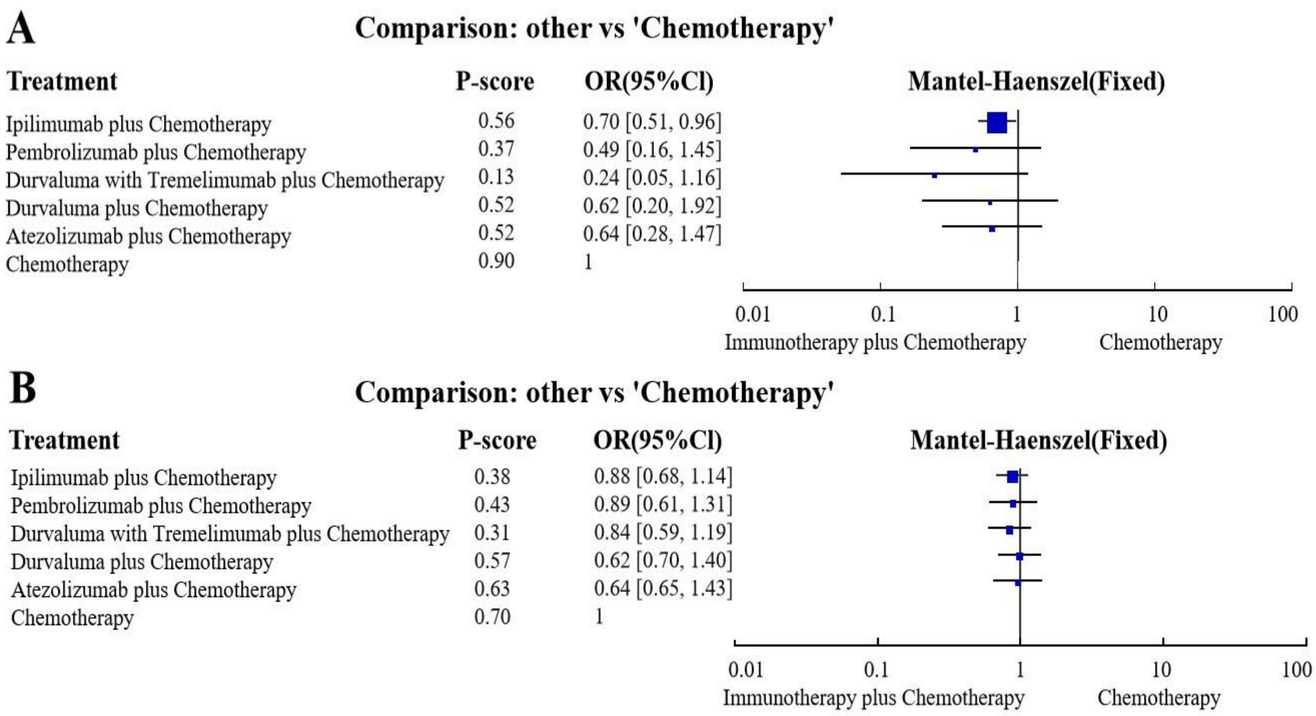

Abbreviation: OR, odds ratio.

The forest plots of any AEs (A) and AEs (B) grade 3/4 in the comparisons of five immuno-chemotherapy regimens versus chemotherapy treatment.

eFigure 5. Model structure for cost-effectiveness analysis.

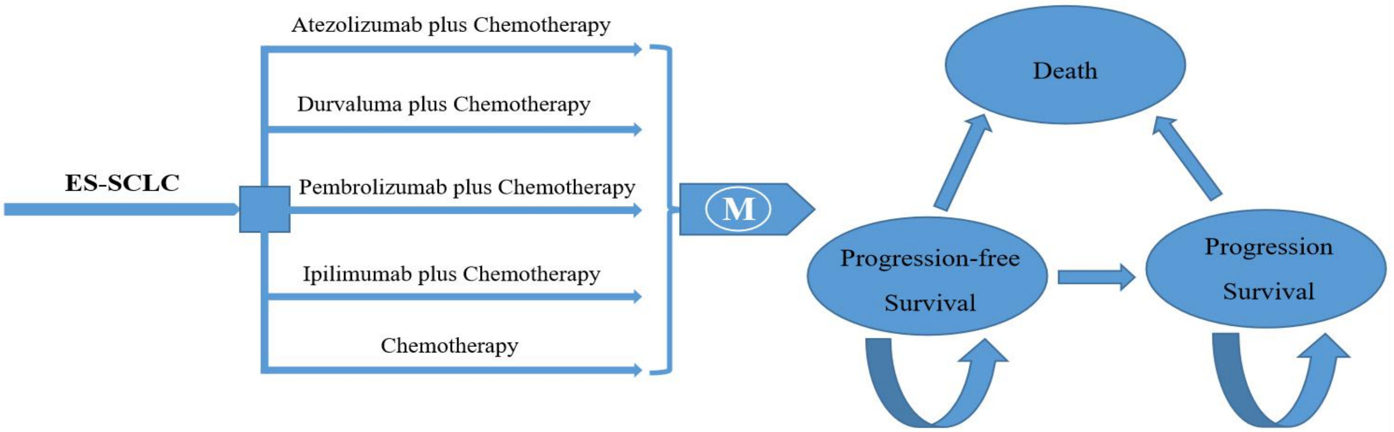

Abbreviation: M, Markov.

eFigure 6. OS Kaplan-Meier Curve Fitting and Extrapolation.

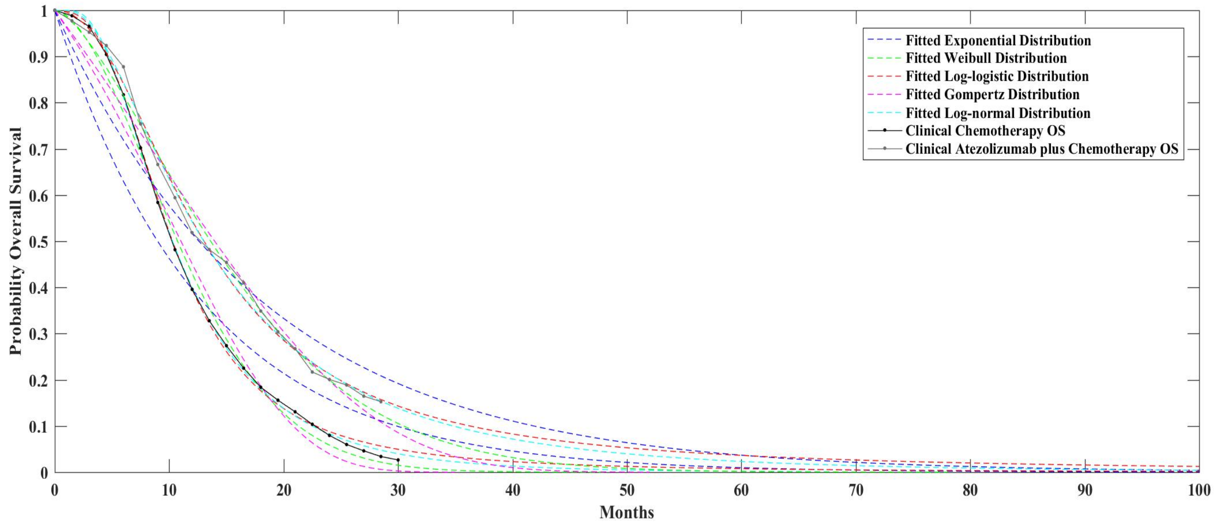

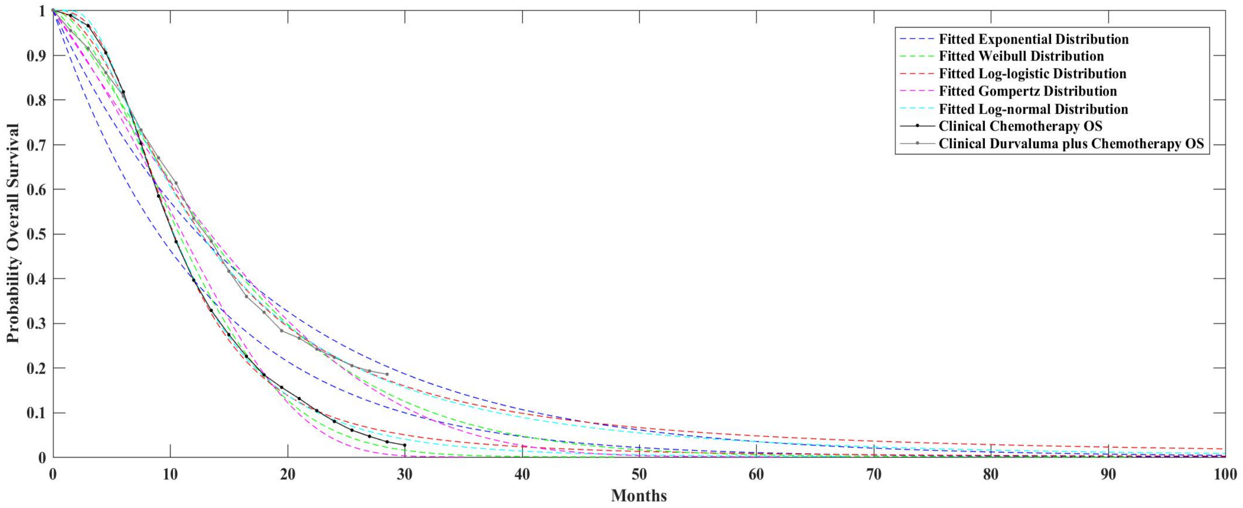
Fitted Exponential Distribution
Fitted Weibull Distribution
Fitted Log-logistic Distribution
Fitted Gompertz Distribution
Fitted Log-normal Distribution
Clinical Chemotherapy OS
Clinical Durvaluma plus Chemotherapy OS
Probability Overall Survival
Months

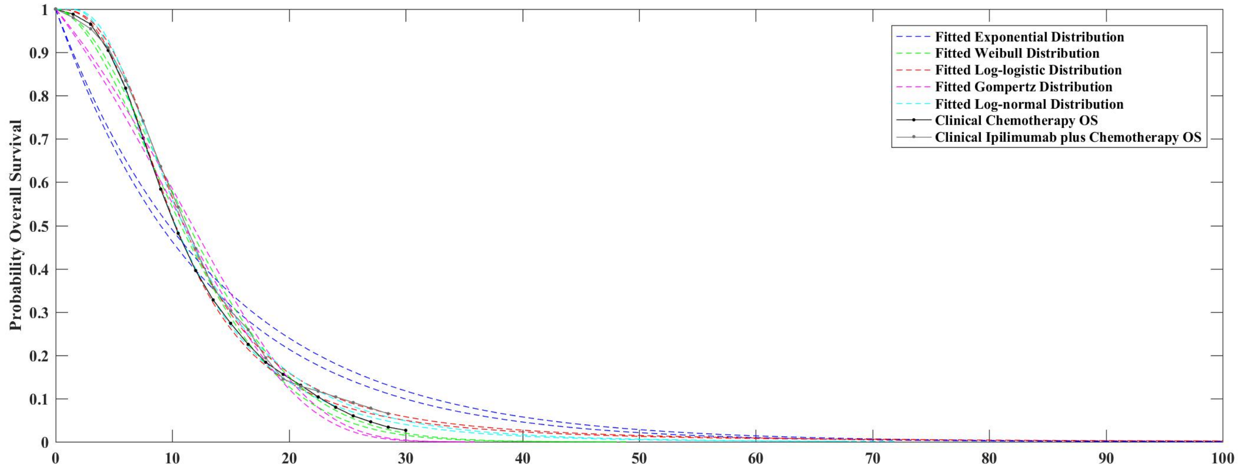
Fitted Exponential Distribution
Fitted Weibull Distribution
Fitted Log-logistic Distribution
Fitted Gompertz Distribution
Fitted Log-normal Distribution
Clinical Chemotherapy OS
Clinical Ipilimumab plus Chemotherapy OS
Probability Overall Survival

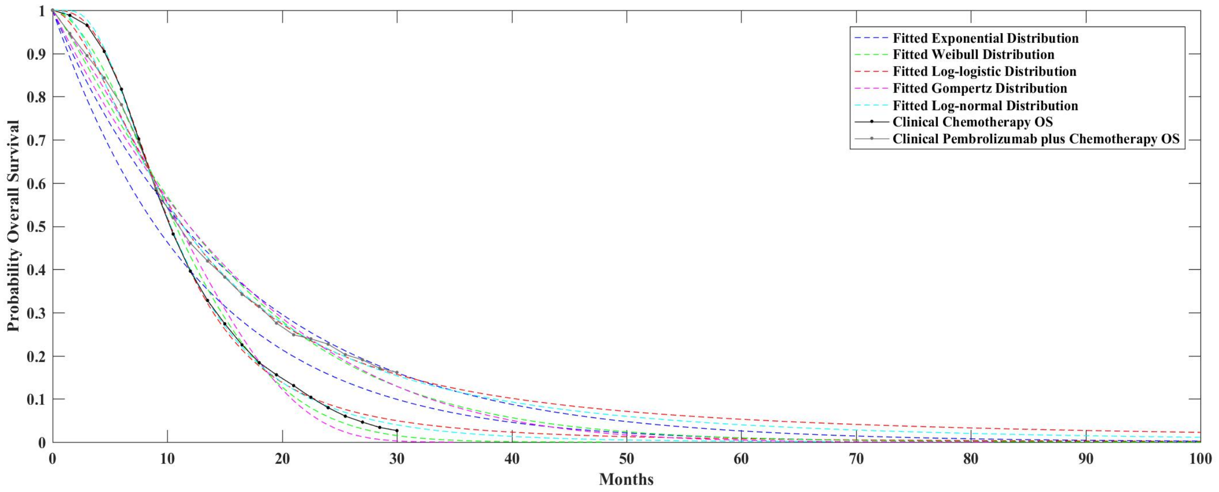
Fitted Exponential Distribution
Fitted Weibull Distribution
Fitted Log-logistic Distribution
Fitted Gompertz Distribution
Fitted Log-normal Distribution
Clinical Chemotherapy OS
Clinical Pembrolizumab plus Chemotherapy OS
Probability Overall Survival
Months

eFigure 7. PFS Kaplan-Meier Curve Fitting and Extrapolation.

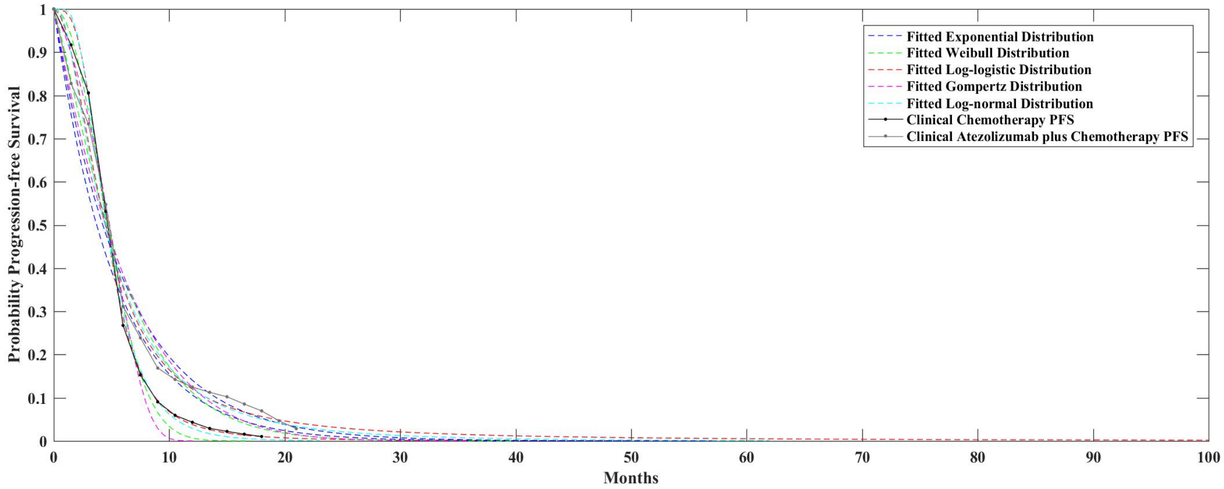

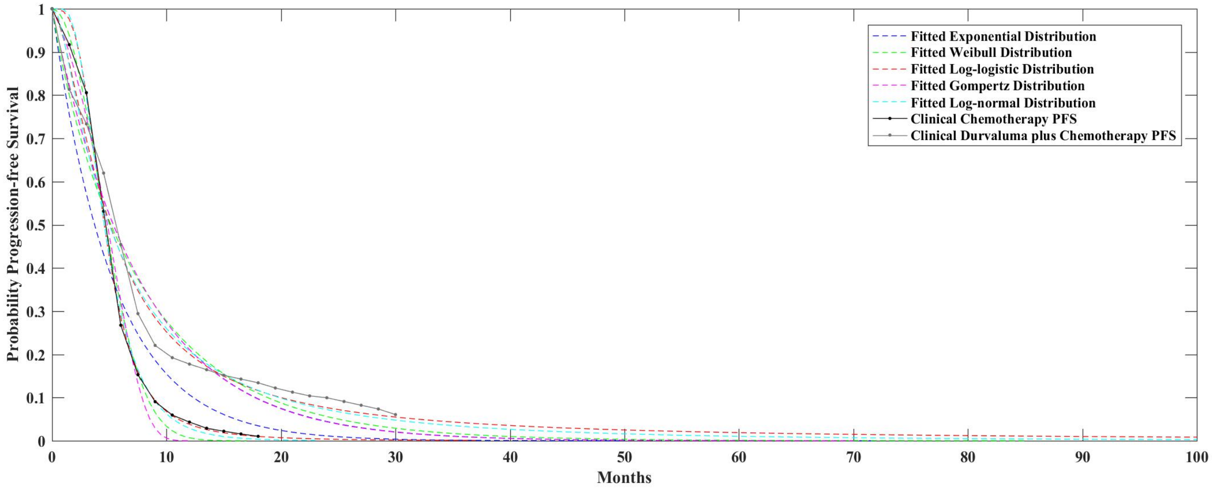

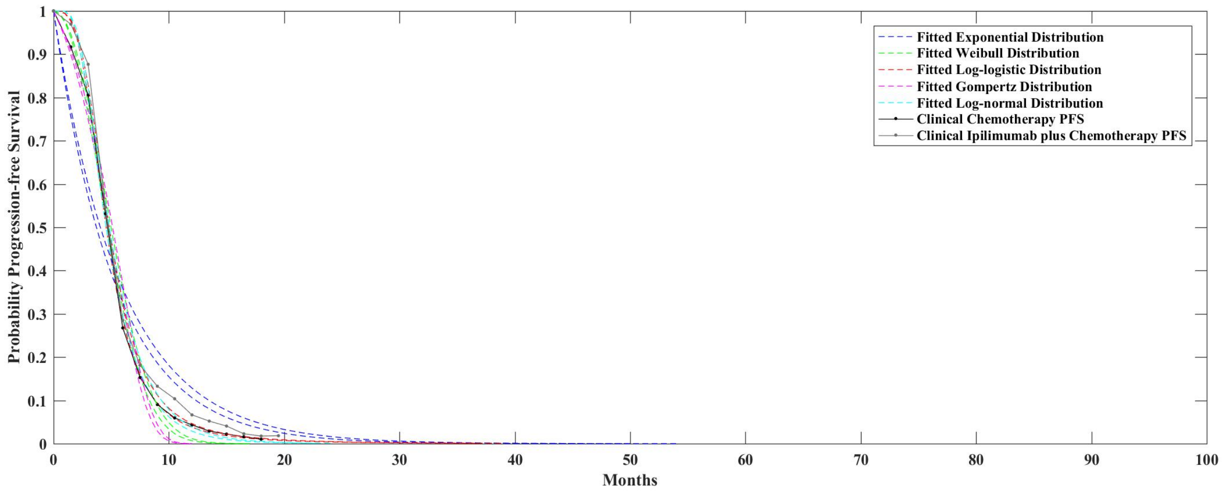

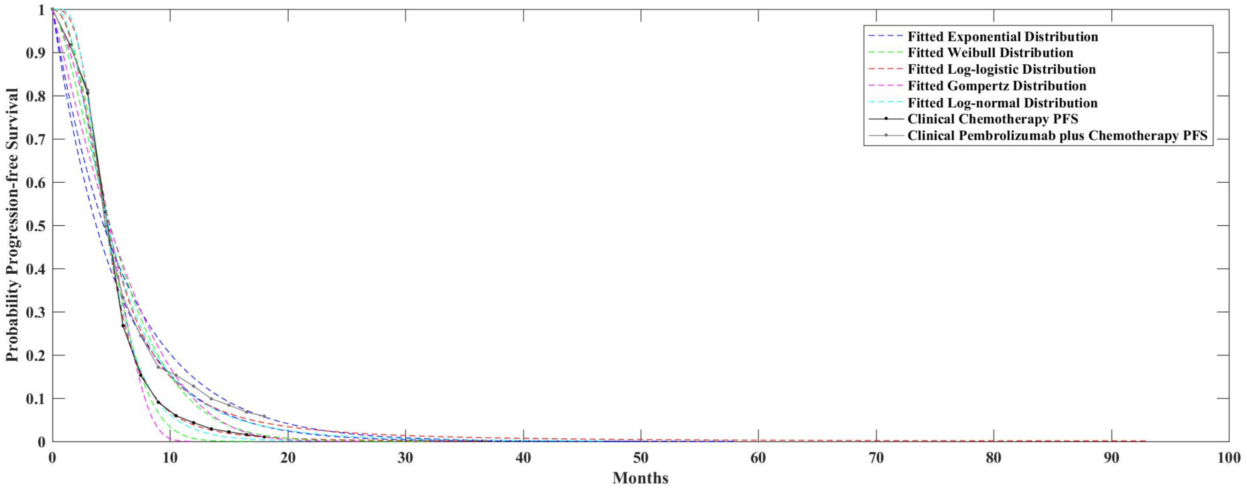

eFigure 8. The one-way sensitivity analyses.

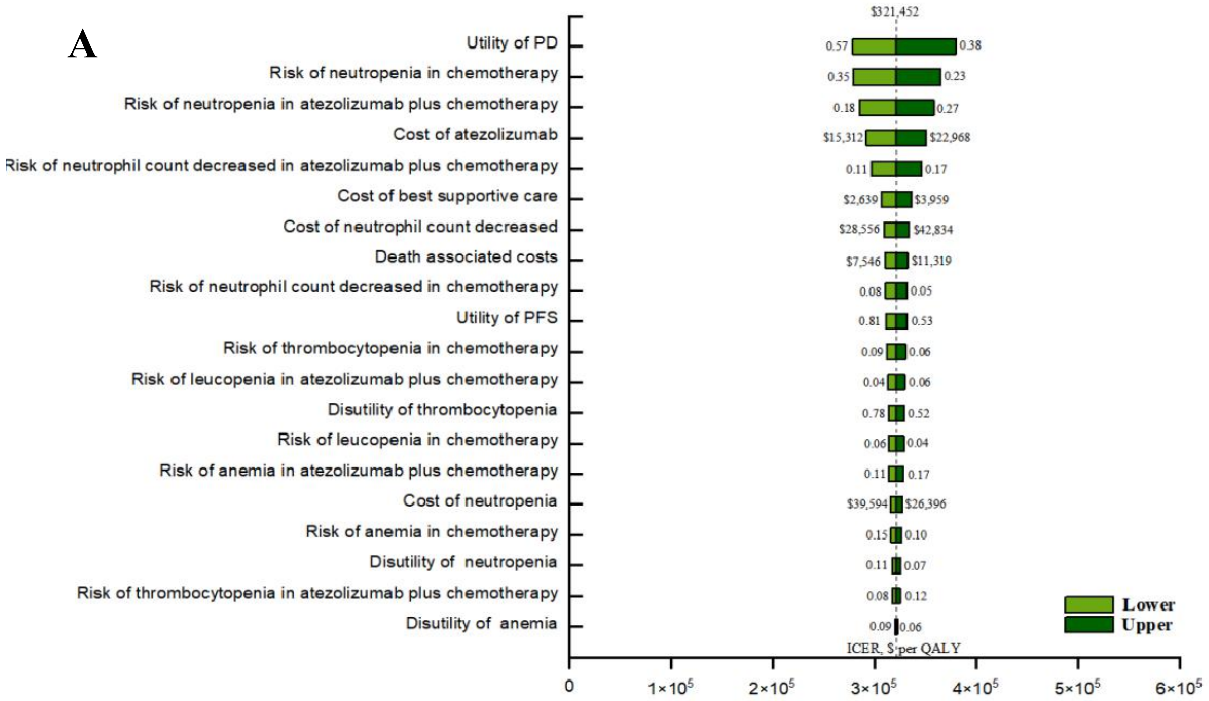

B

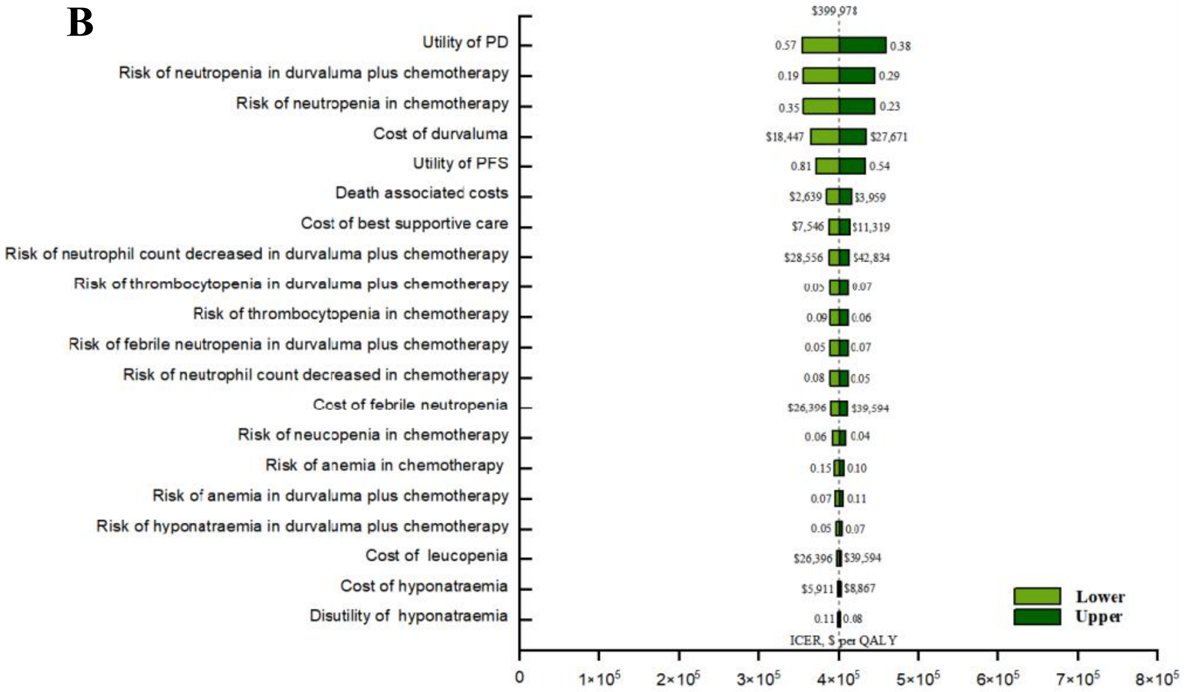
$399,978
Utility of PD
Risk of neutropenia in durvaluma plus chemotherapy
Risk of neutropenia in chemotherapy
Cost of durvaluma
Utility of PFS
Death associated costs
Cost of best supportive care
Risk of neutrophil count decreased in durvaluma plus chemotherapy
Risk of thrombocytopenia in durvaluma plus chemotherapy
Risk of thrombocytopenia in chemotherapy
Risk of febrile neutropenia in durvaluma plus chemotherapy
Risk of neutrophil count decreased in chemotherapy
Cost of febrile neutropenia
Risk of neucopenia in chemotherapy
Risk of anemia in chemotherapy
Risk of anemia in durvaluma plus chemotherapy
Risk of hyponatraemia in durvaluma plus chemotherapy
Cost of leucopenia
Cost of hyponatraemia
Disutility of hyponatraemia
0.57 0.38
0.19 0.29
0.35 0.23
$18,447 $27,671
0.81 0.54
$2,639 $3,959
$7,546 $11,319
$28,556 $42,834
0.05 0.07
0.09 0.06
0.05 0.07
0.08 0.05
$26,396 $39,594
0.06 0.04
0.15 0.10
0.07 0.11
0.05 0.07
$26,396 $39,594
$5,911 $8,867
0.11 0.08
Lower
Upper
ICER, $ per QALY
0
1×10^5
2×10^5
3×10^5
4×10^5
5×10^5
6×10^5
7×10^5
8×10^5

C

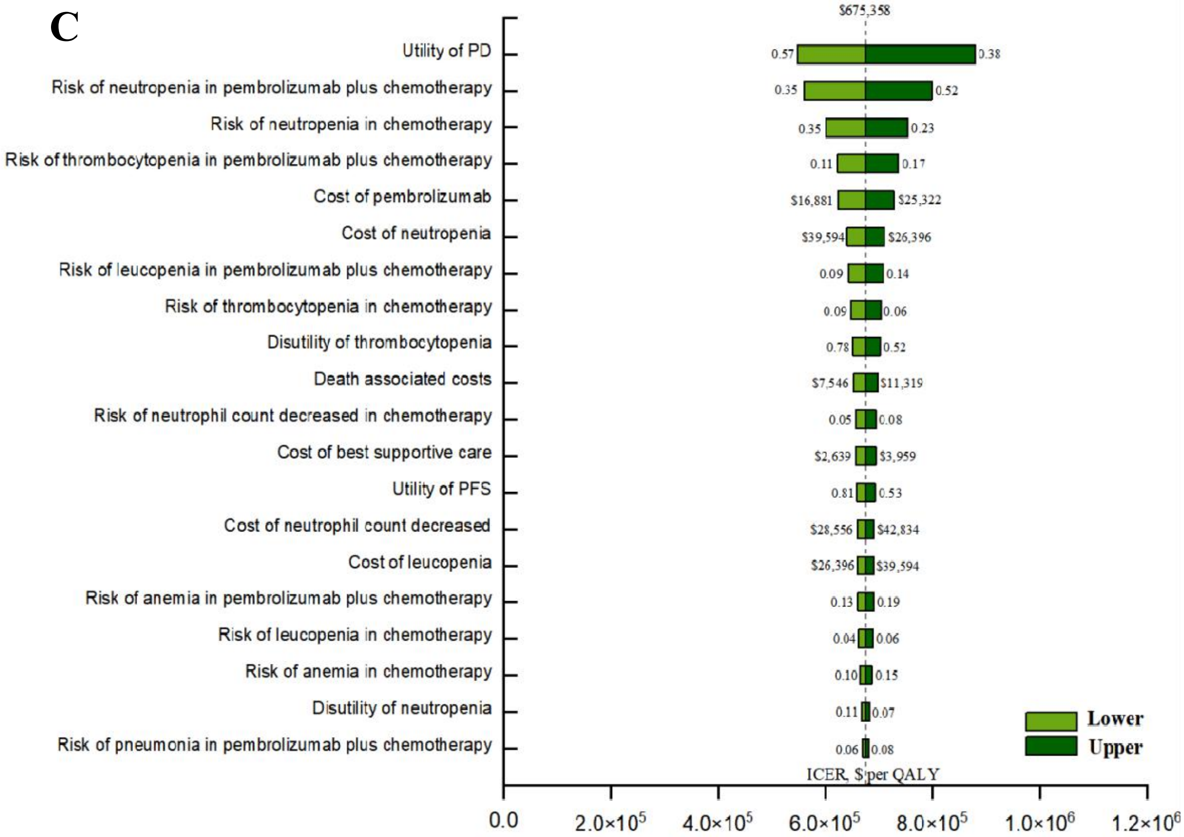
$675,358
Utility of PD
Risk of neutropenia in pembrolizumab plus chemotherapy
Risk of neutropenia in chemotherapy
Risk of thrombocytopenia in pembrolizumab plus chemotherapy
Cost of pembrolizumab
Cost of neutropenia
Risk of leucopenia in pembrolizumab plus chemotherapy
Risk of thrombocytopenia in chemotherapy
Disutility of thrombocytopenia
Death associated costs
Risk of neutrophil count decreased in chemotherapy
Cost of best supportive care
Utility of PFS
Cost of neutrophil count decreased
Cost of leucopenia
Risk of anemia in pembrolizumab plus chemotherapy
Risk of leucopenia in chemotherapy
Risk of anemia in chemotherapy
Disutility of neutropenia
Risk of pneumonia in pembrolizumab plus chemotherapy
0.57 0.38
0.35 0.52
0.35 0.23
0.11 0.17
$16,881 $25,322
$39,594 $26,396
0.09 0.14
0.09 0.06
0.78 0.52
$7,546 $11,319
0.05 0.08
$2,639 $3,959
0.81 0.53
$28,556 $42,834
$26,396 $39,594
0.13 0.19
0.04 0.06
0.10 0.15
0.11 0.07
0.06 0.08
Lower
Upper
ICER, $ per QALY
0.0
2.0×10^5
4.0×10^5
6.0×10^5
8.0×10^5
1.0×10^6
1.2×10^6

D

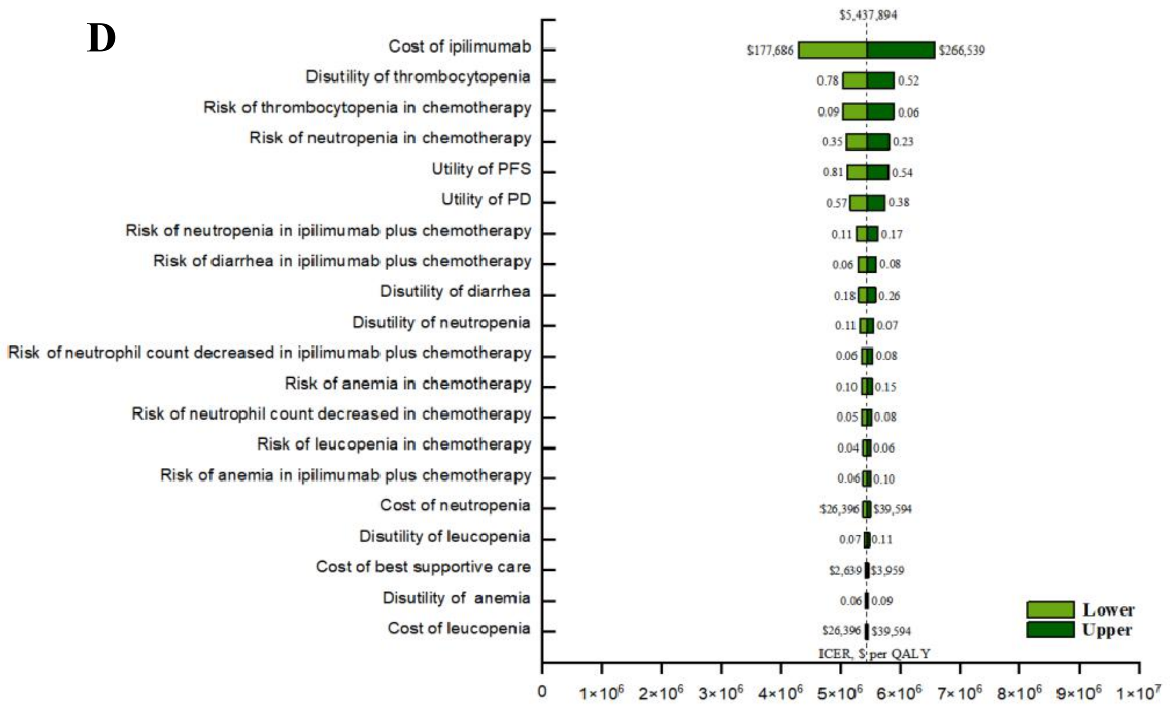

Abbreviation: PFS, progression-free survival; PD, progressive disease; ICER, incremental cost-effectiveness ratio; QALY, quality-adjusted life-year.

The one-way sensitivity analyses of atezolizumab plus chemotherapy (A), durvalumab plus chemotherapy (B), pembrolizumab plus chemotherapy (C), ipilimumab plus chemotherapy (D) in comparison with chemotherapy.

eFigure 9. Probability Sensitivity Analysis Scatter Plot of the overall populations.

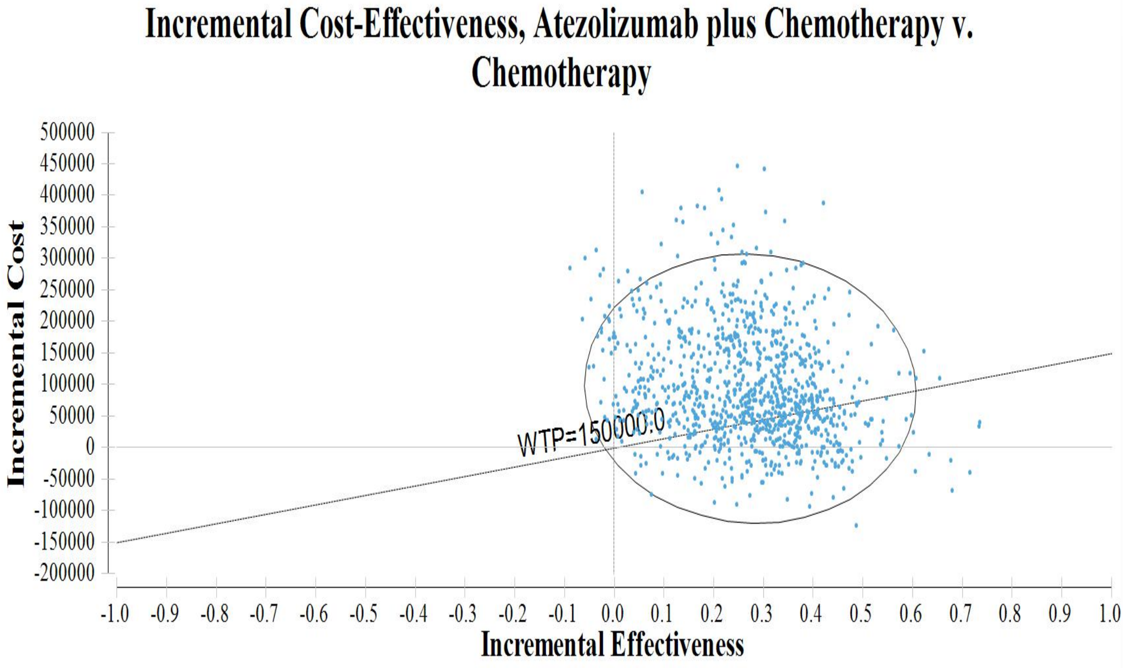

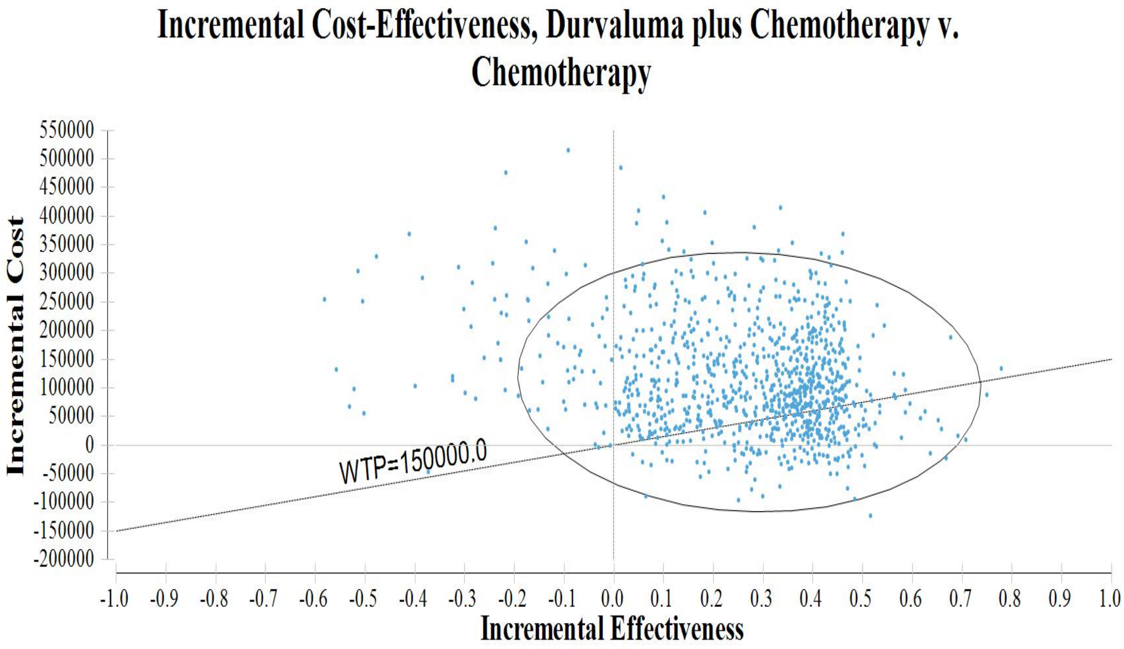
Incremental Cost-Effectiveness, Durvaluma plus Chemotherapy v. Chemotherapy
Incremental Cost
Incremental Effectiveness
WTP=150000.0
550000
500000
450000
400000
350000
300000
250000
200000
150000
100000
50000
0
-50000
-100000
-150000
-200000
-1.0
-0.9
-0.8
-0.7
-0.6
-0.5
-0.4
-0.3
-0.2
-0.1
0.0
0.1
0.2
0.3
0.4
0.5
0.6
0.7
0.8
0.9
1.0

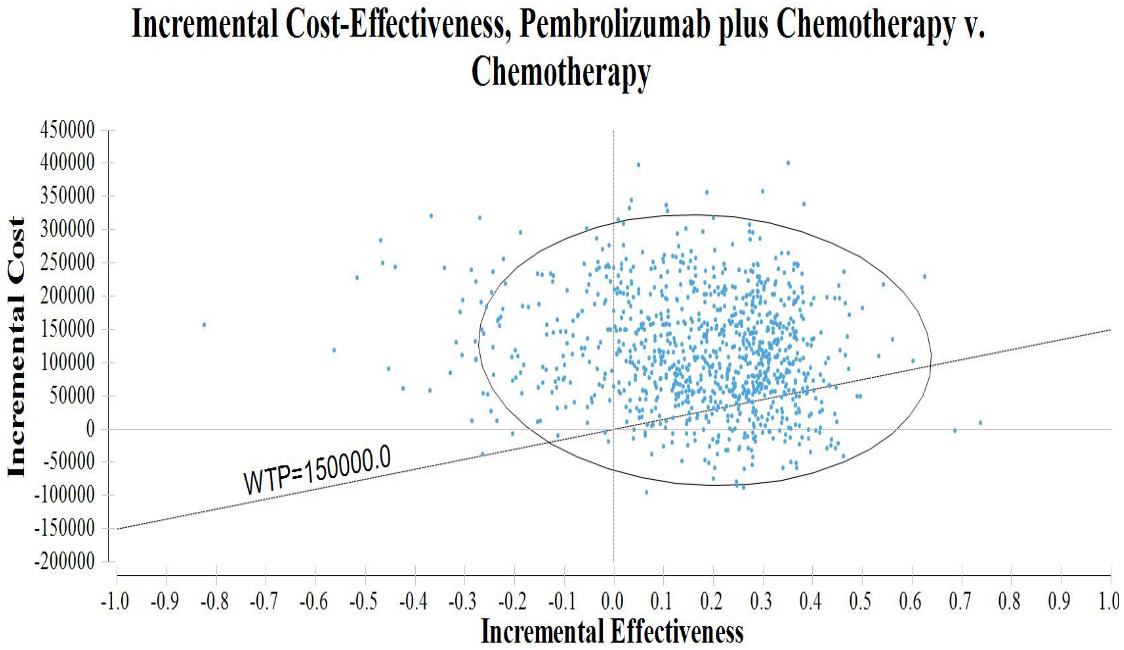
Incremental Cost-Effectiveness, Pembrolizumab plus Chemotherapy v. Chemotherapy
Incremental Cost
Incremental Effectiveness
WTP=150000.0
450000
400000
350000
300000
250000
200000
150000
100000
50000
0
-50000
-100000
-150000
-200000
-1.0
-0.9
-0.8
-0.7
-0.6
-0.5
-0.4
-0.3
-0.2
-0.1
0.0
0.1
0.2
0.3
0.4
0.5
0.6
0.7
0.8
0.9
1.0

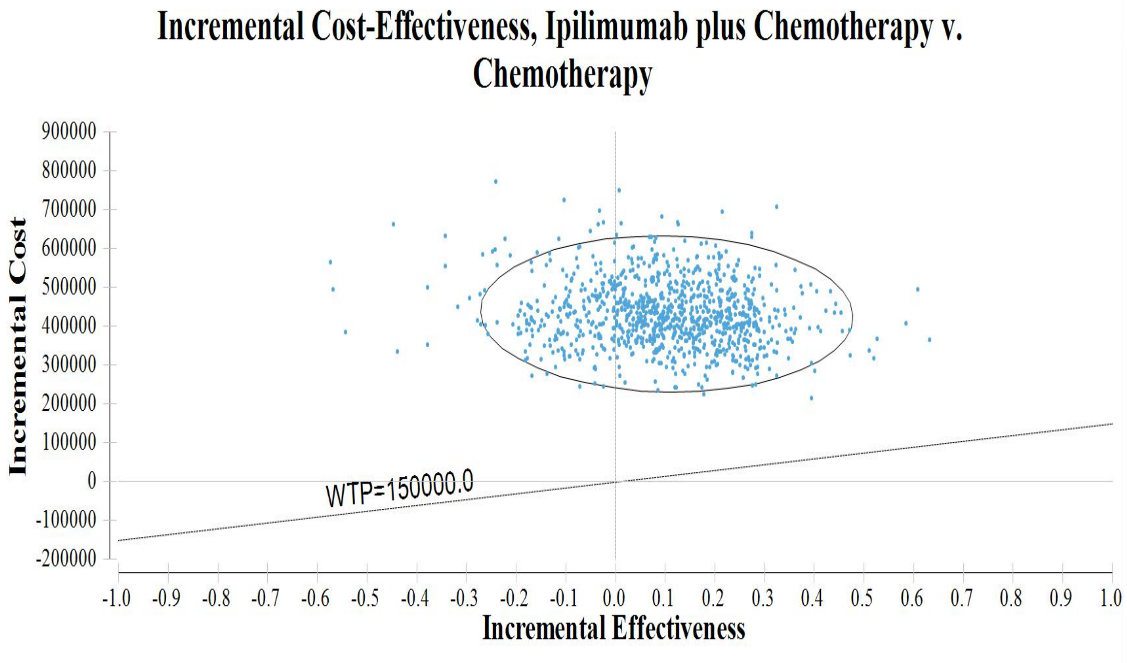

eFigure 10. Probability Sensitivity Analysis Scatter Plot of the population with brain metastases.

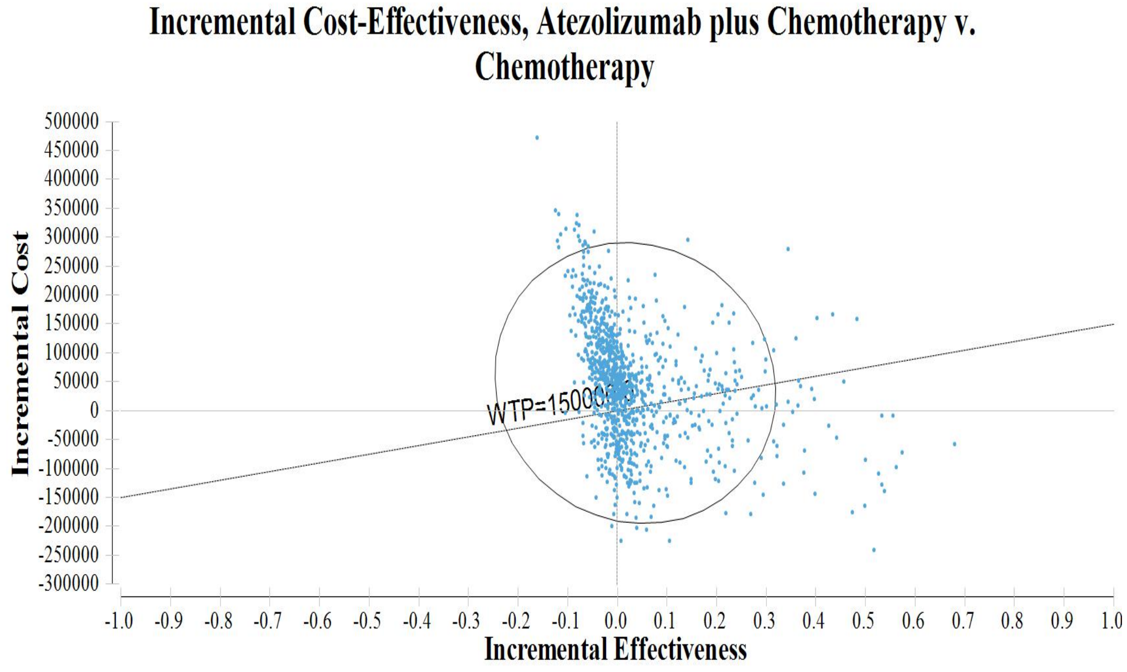

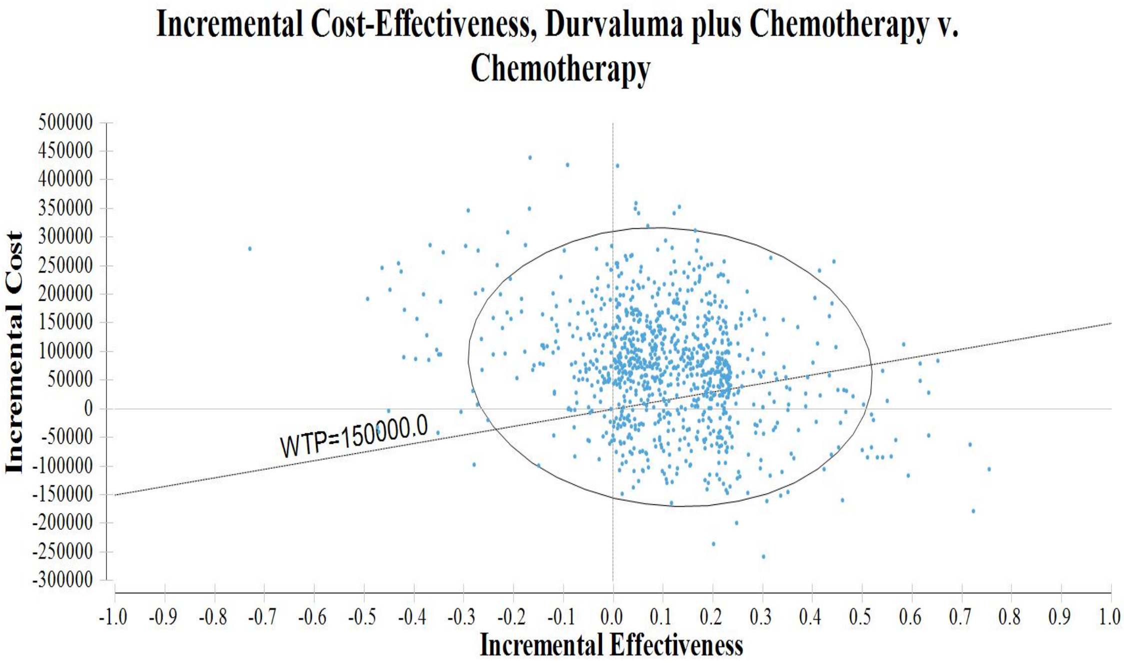
Incremental Cost-Effectiveness, Durvaluma plus Chemotherapy v. Chemotherapy
Incremental Cost
Incremental Effectiveness
WTP=150000.0
500000
450000
400000
350000
300000
250000
200000
150000
100000
50000
0
-50000
-100000
-150000
-200000
-250000
-300000
-1.0 -0.9 -0.8 -0.7 -0.6 -0.5 -0.4 -0.3 -0.2 -0.1 0.0 0.1 0.2 0.3 0.4 0.5 0.6 0.7 0.8 0.9 1.0

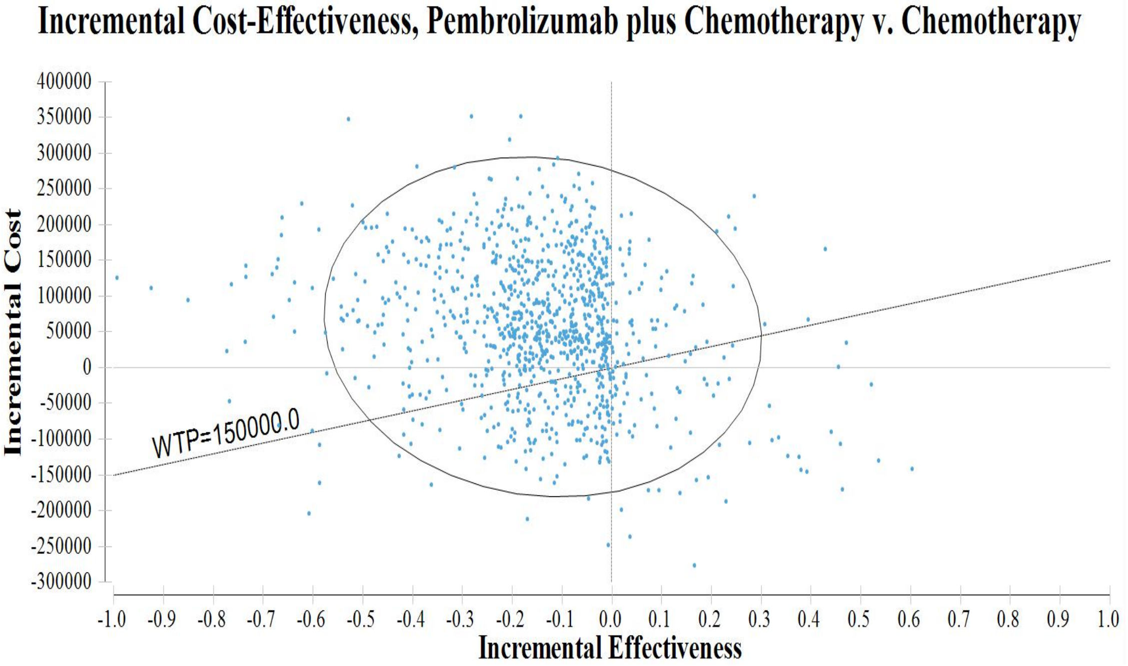
Incremental Cost-Effectiveness, Pembrolizumab plus Chemotherapy v. Chemotherapy
Incremental Cost
Incremental Effectiveness
WTP=150000.0
400000
350000
300000
250000
200000
150000
100000
50000
0
-50000
-100000
-150000
-200000
-250000
-300000
-1.0 -0.9 -0.8 -0.7 -0.6 -0.5 -0.4 -0.3 -0.2 -0.1 0.0 0.1 0.2 0.3 0.4 0.5 0.6 0.7 0.8 0.9 1.0

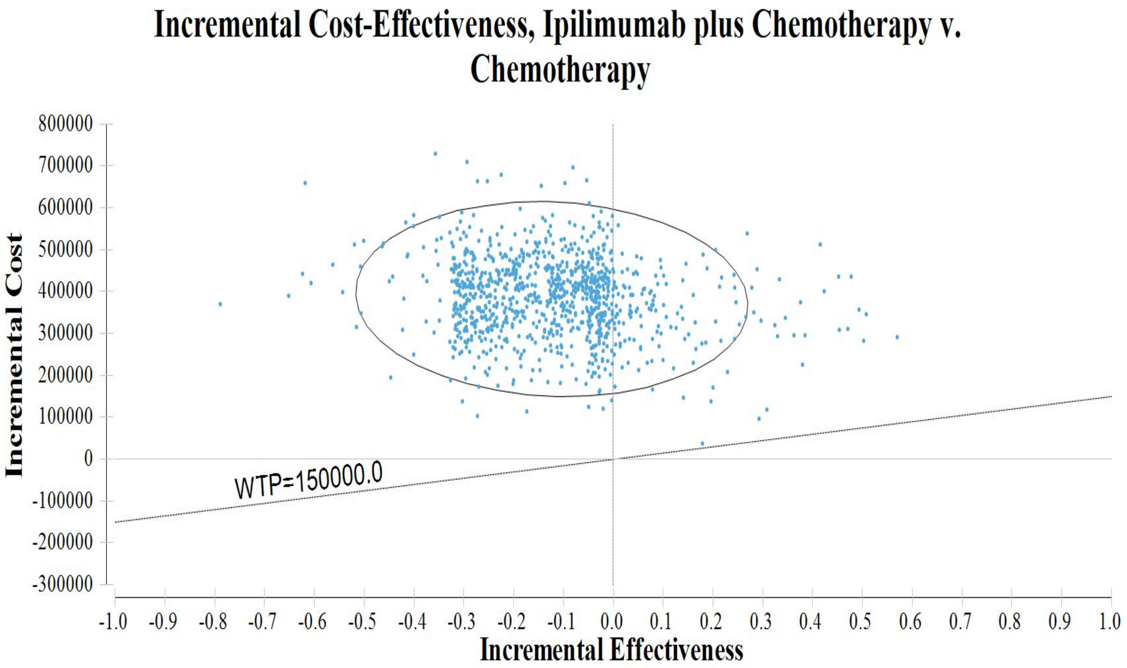

eFigure 11. Probability Sensitivity Analysis Scatter Plot of the population with non-brain metastases.

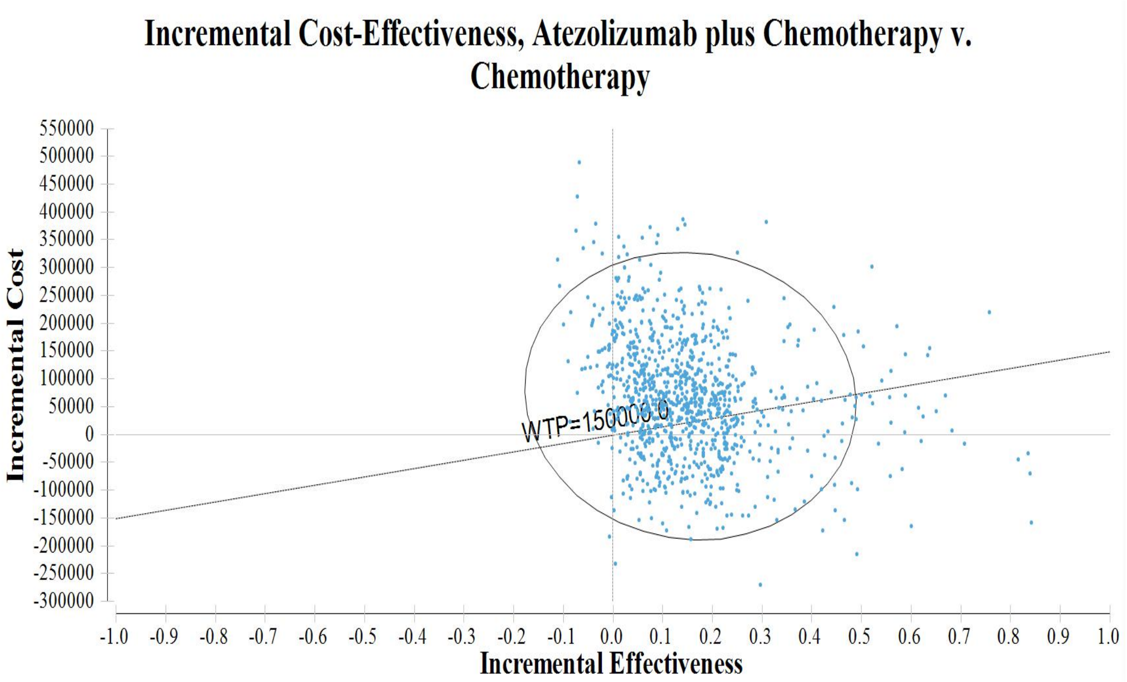

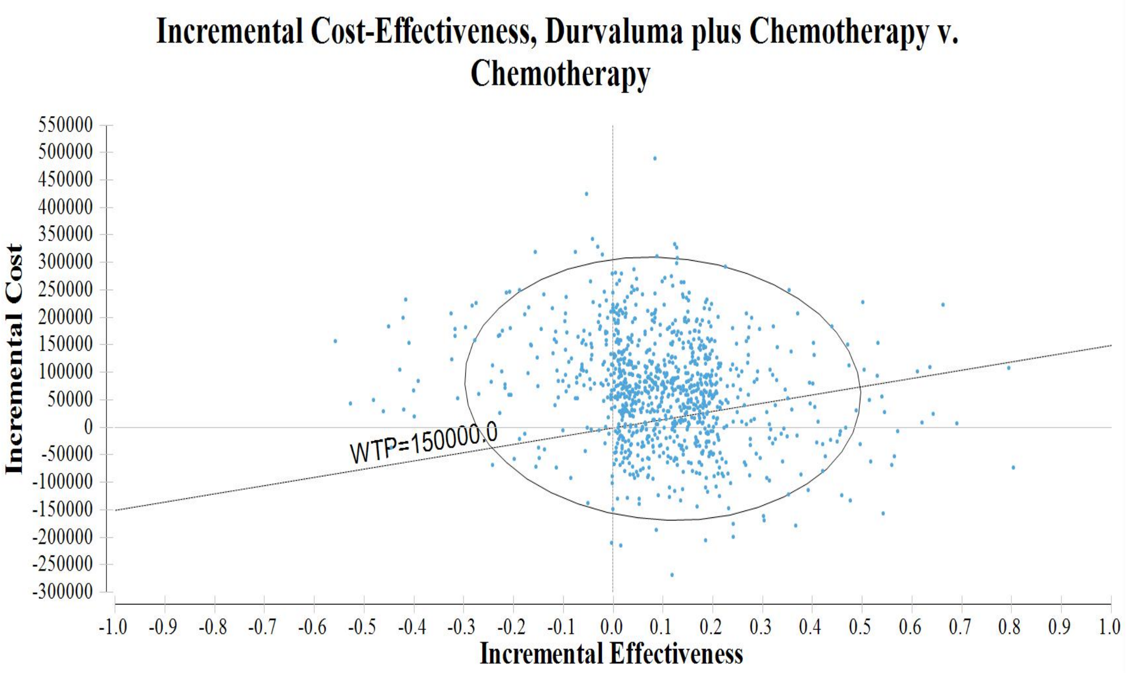
Incremental Cost-Effectiveness, Durvaluma plus Chemotherapy v. Chemotherapy
Incremental Cost
550000
500000
450000
400000
350000
300000
250000
200000
150000
100000
50000
0
-50000
-100000
-150000
-200000
-250000
-300000
WTP=150000.0
-1.0 -0.9 -0.8 -0.7 -0.6 -0.5 -0.4 -0.3 -0.2 -0.1 0.0 0.1 0.2 0.3 0.4 0.5 0.6 0.7 0.8 0.9 1.0
Incremental Effectiveness

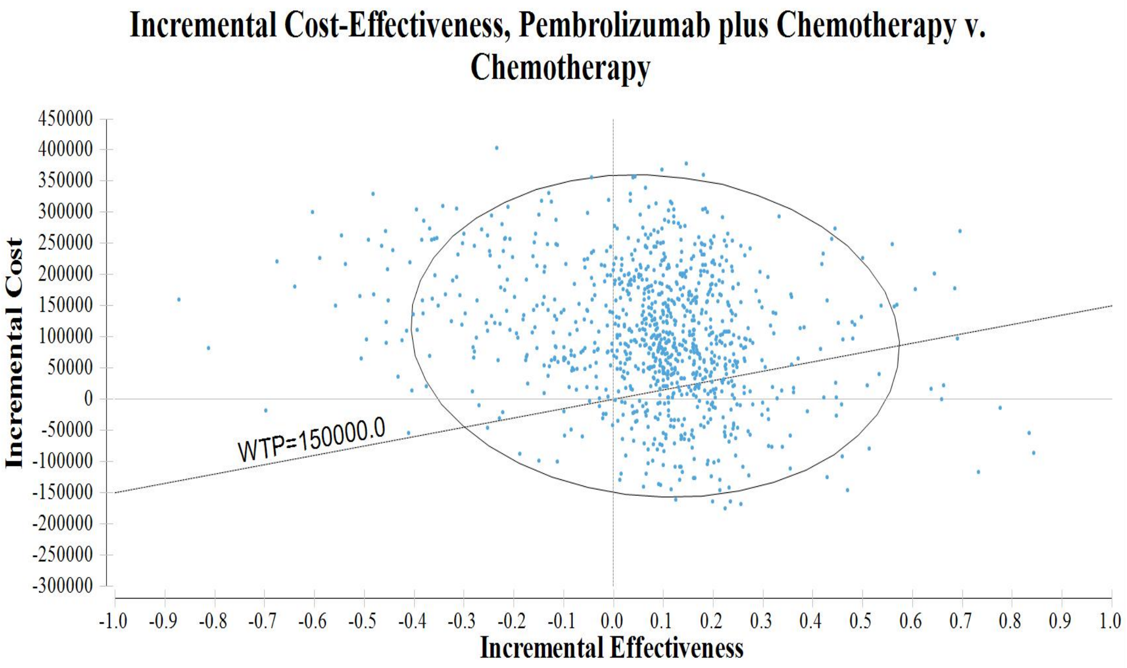
Incremental Cost-Effectiveness, Pembrolizumab plus Chemotherapy v. Chemotherapy
Incremental Cost
450000
400000
350000
300000
250000
200000
150000
100000
50000
0
-50000
-100000
-150000
-200000
-250000
-300000
WTP=150000.0
-1.0 -0.9 -0.8 -0.7 -0.6 -0.5 -0.4 -0.3 -0.2 -0.1 0.0 0.1 0.2 0.3 0.4 0.5 0.6 0.7 0.8 0.9 1.0
Incremental Effectiveness

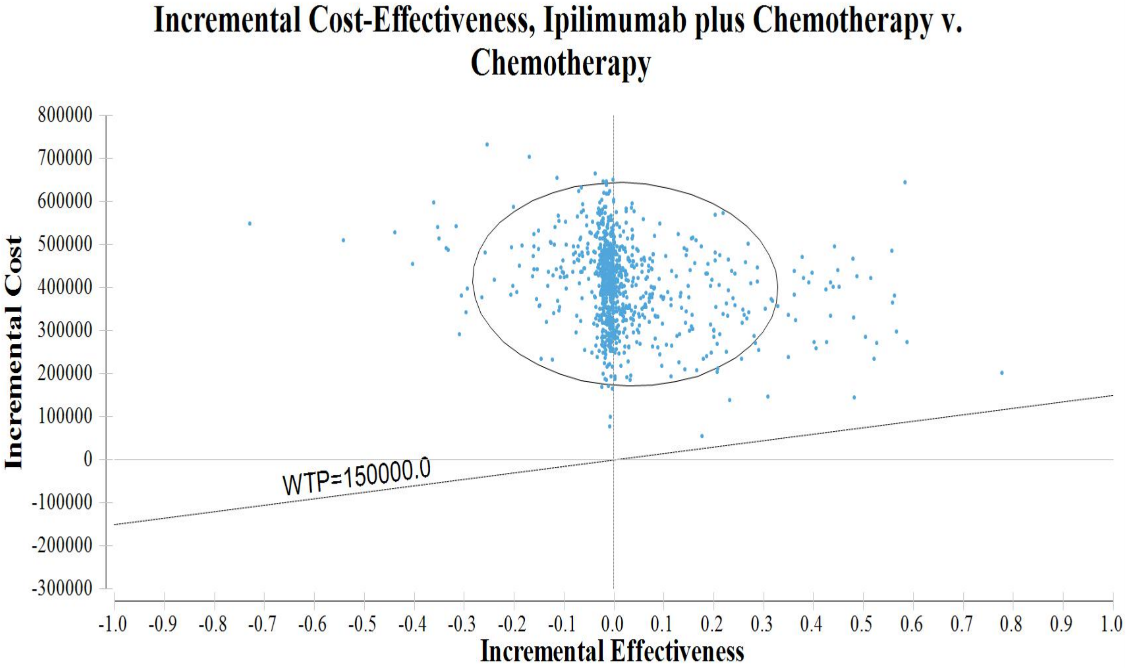

Abbreviation: WTP, willingness-to-pay.

Each point in the diagram represents a simulation result of 10,000 Monte Carlo simulation. Ellipse represent the 95% CI and dotted line represent WTP ($150,000/QALY). Points to the right of the dotted line are considered cost-effective.

eTable 1. Search strategy.

Database	Keywords
**Pubmed**	
**(1)**	"nivolumab"[Title/Abstract] OR "pembrolizumab"[Title/Abstract] OR "‘ipilimumab" [Title/Abstract] OR "atezolizumab"[Title/Abstract] OR "camrelizumab"[Title/Abstract] OR "cemiplimab"[Title/Abstract] OR "durvalumab"[Title/Abstract] OR "toripalimab"[Title/Abstract] OR "tislelizumab"[Title/Abstract] OR "PD-1"[Title/Abstract] OR "PD-L1"[Title/Abstract] OR "anti-PD-1"[Title/Abstract] OR "anti-PD-L1"[Title/Abstract] OR "Immune checkpoint inhibitor"[Title/Abstract] OR "ICIs "[Title/Abstract] OR "programmed cell death 1 receptor/antagonists and inhibitors"[MeSH Terms] OR "programmed cell death 1 receptor antagonists and inhibitors"[Title/Abstract] OR "programmed cell death 1 receptor antagonist"[Title/Abstract] OR "programmed cell death 1 receptor inhibitor"[Title/Abstract] OR "Immunotherapy"[Title/Abstract]
**(2)**	"platinum"[Title/Abstract] OR "cisplatin"[Title/Abstract] OR "carboplatin"[Title/Abstract] OR "nedaplatin"[Title/Abstract] OR "etoposide"[Title/Abstract] OR "irinotecan"[Title/Abstract] OR "amrubicin"[Title/Abstract] OR "chemoptherapy"[Title/Abstract]
**(3)**	"Small Cell Lung Cancer"[Title/Abstract] OR "Small Cell Lung Carcinoma"[MeSH Terms] OR "Small-Cell Lung Carcinoma"[Title/Abstract] OR "Small-Cell Lung Carcinoma"[Title/Abstract] OR "SCLC"[Title/Abstract]
**(4)**	"Extensive-stage"[Title/Abstract] OR "Extensive stage"[Title/Abstract] OR "Extensive-disease"[Title/Abstract] OR "Extensive disease"[Title/Abstract] OR "advanced"[Title/Abstract] OR "extensive"[Title/Abstract]
**(5)**	"clinical trials as topic"[MeSH Terms] OR "clinical trial"[Publication Type] OR "phase III"[Title/Abstract] OR "phase 3"[Title/Abstract] OR "clinical trials"[Title/Abstract]
**(6)**	("2000/01/01"[Date - Publication] : "2020/11/30"[Date - Publication])
**(7)**	(1) AND (2) AND (3) AND (4) AND (5) AND (6)
**(8)**	"Carcinoma, Non-Small-Cell Lung"[MeSH Terms] OR "non small cell lung cancer"[Title/Abstract] OR "non-small cell lung cancer"[Title/Abstract] OR "non-small-cell lung cancer"[Title/Abstract] OR "NSCLC"[Title/Abstract]
**(9)**	"review"[Article type] OR "meta"[Title] OR "meta-analysis"[Title] OR "protocol"[Title]
**(10)**	(7) NOT (8) NOT (9)
**Embase**	
**(1)**	(nivolumab OR pembrolizumab OR ipilimumab OR atezolizumab OR camrelizumab OR OR cemiplimab OR durvalumab OR toripalimab OR tislelizumab OR tislelizumab OR PD-1 OR PD-L1 OR anti-PD-1 OR anti-PD-1 OR anti-PD-L1 OR 'immune checkpoint inhibitors' OR ICIs OR 'programmed cell death 1 receptor/antagonists and inhibitors' OR 'programmed cell death 1 receptor antagonists and inhibitors' OR 'programmed cell death 1 receptor antagonist' OR 'programmed cell death 1 receptor inhibitor' OR Immunotherapy):ti,ab,kw
**(2)**	(platinum OR cisplatin OR carboplatin OR nedaplatin OR etoposide OR irinotecan OR amrubicin OR chemoptheray):ti,ab,kw

**(3)**	('Small Cell Lung Cancer' OR 'Small Cell Lung Carcinoma' OR 'Small-Cell Lung Carcinoma' OR 'Small-Cell Lung Carcinoma' OR 'SCLC'):ti,ab,kw
**(4)**	('Extensive-stage' OR 'Extensive stage' OR 'Extensive-disease' OR 'Extensive disease' OR advanced OR extensive):ti,ab,kw
**(5)**	trial/exp OR 'clinical trials'/exp OR 'phase 3 clinical trial'/exp OR 'phase III clinical trial'/exp
**(6)**	[article]/lim OR [article in press]/lim
**(7)**	(1) AND (2) AND (3) AND (4) AND (5) AND (6) AND (7)
**(8)**	("Carcinoma, Non-Small-Cell Lung" OR "non small cell lung cancer" OR "non-small cell lung cancer" OR "non-small-cell lung cancer" OR "NSCLC"):ti,ab,kw
**(9)**	(8) AND [1-1-2000]/sd NOT [30-11-2021]/sd
**(10)**	(10) NOT (9)
**Cochrane**	
**(1)**	(nivolumab OR pembrolizumab OR ipilimumab OR atezolizumab OR camrelizumab OR cemiplimab OR durvalumab OR toripalimab OR tislelizumab OR tislelizumab OR PD-1 OR PD-L1 OR anti-PD-1 OR anti-PD-1 OR anti-PD-L1 OR 'immune checkpoint inhibitors' OR ICIs OR 'programmed cell death 1 receptor and antagonists and inhibitors' OR 'programmed cell death 1 receptor antagonists and inhibitors' OR 'programmed cell death 1 receptor antagonist' OR 'programmed cell death 1 receptor inhibitor' OR immunotherapy)
**(2)**	(platinum OR cisplatin OR carboplatin OR nedaplatin OR etoposide OR irinotecan OR amrubicin OR chemoptherapy)
**(3)**	('Small Cell Lung Cancer' OR 'Small Cell Lung Carcinoma' OR 'Small-Cell Lung Cancer' OR 'Small-Cell Lung Carcinoma' OR SCLC)
**(4)**	('Extensive-stage' OR 'Extensive stage' OR Extensive-disease OR 'Extensive disease' OR advanced OR extensive)
**(5)**	('clinical trials as topic' OR trial):ti,ab,kw
**(6)**	("conference" OR "review"):pt
**(7)**	("Carcinoma, Non-Small-Cell Lung" OR "non small cell lung cancer" OR "non-small cell lung cancer" OR "non-small-cell lung cancer" OR "NSCLC"):ti,ab,kw
**(8)**	(1) AND (2) AND (3) AND (4) AND (5) NOT (6) NOT (7)
**(9)**	Publication date: Between Jan 2000 and Nov 2021
**(10)**	(8) AND (9)
**Web of science**	
**(1)**	TS=(nivolumab OR pembrolizumab OR ipilimumab OR atezolizumab OR camrelizumab OR cemiplimab OR durvalumab OR toripalimab OR tislelizumab OR tislelizumab OR PD-1 OR PD-L1 OR anti-PD-1 OR anti-PD-1 OR anti-PD-L1 OR 'immune checkpoint inhibitors' OR ICIs OR 'programmed cell death 1 receptor and antagonists and inhibitors' OR 'programmed cell death 1 receptor antagonists and inhibitors' OR 'programmed cell death 1 receptor antagonist' OR 'programmed cell death 1 receptor inhibitor' OR immunotherapy)

**(2)**	TS=(nivolumab OR pembrolizumab OR ipilimumab OR atezolizumab OR camrelizumab OR cemiplimab OR durvalumab OR toripalimab OR tislelizumab OR tislelizumab OR PD-1 OR PD-L1 OR anti-PD-1 OR anti-PD-1 OR anti-PD-L1 OR 'immune checkpoint inhibitors' OR ICIs OR 'programmed cell death 1 receptor and antagonists and inhibitors' OR 'programmed cell death 1 receptor antagonists and inhibitors' OR 'programmed cell death 1 receptor antagonist' OR 'programmed cell death 1 receptor inhibitor' OR immunotherapy)
**(3)**	TS=('Small Cell Lung Cancer' OR 'Small Cell Lung Carcinoma' OR 'Small-Cell Lung Cancer' OR 'Small-Cell Lung Carcinoma' OR SCLC)
**(4)**	TS=('Extensive-stage' OR 'Extensive stage' OR Extensive-disease OR 'Extensive disease' OR advanced OR extensive)
**(5)**	TS=("clinical trials as topic"OR "trial" OR "phase III" OR "phase 3" OR "clinical trials")
**(6)**	TS=("conference" OR "review" OR "meta")
**(7)**	TS=("Carcinoma, Non-Small-Cell Lung" OR "non small cell lung cancer" OR "non-small cell lung cancer" OR "non-small-cell lung cancer" OR "NSCLC")
**(8)**	(1) AND (2) AND (3) AND (4) AND (5)
**(9)**	NOT (6) NOT (7) (Date range: publication date: 2000-01-01—2021-11-30)

eTable 2. PRISMA NMA Checklist.

Section/Topic	Item #	Checklist Item	Reported on Page #
**TITLE**			
Title	1	Identify the report as a systematic review *incorporating a network meta-analysis (or related form of meta-analysis).*	1
**ABSTRACT**			
Structured summary	2	Provide a structured summary including, as applicable:<br>**Background:** main objectives<br>**Methods:** data sources; study eligibility criteria, participants, and interventions; study appraisal; and *synthesis methods, such as network meta-analysis.*<br>**Results:** number of studies and participants identified; summary estimates with corresponding confidence/credible intervals; *treatment rankings may also be discussed. Authors may choose to summarize pairwise comparisons against a chosen treatment included in their analyses for brevity.*<br>**Discussion/Conclusions:** limitations; conclusions and implications of findings.<br>**Other:** primary source of funding; systematic review registration number with registry name.	2-3
**INTRODUCTION**			
Rationale	3	Describe the rationale for the review in the context of what is already known*, including mention of why a network meta-analysis has been conducted.*	3-4
Objectives	4	Provide an explicit statement of questions being addressed, with reference to participants, interventions, comparisons, outcomes, and study design (PICOS).	4
**METHODS**			
Protocol and registration	5	Indicate whether a review protocol exists and if and where it can be accessed (e.g., Web address); and, if available, provide registration information, including registration number.	Not applicable
Eligibility criteria	6	Specify study characteristics (e.g., PICOS, length of follow-up) and report characteristics (e.g., years considered, language, publication status) used as criteria for eligibility, giving rationale. *Clearly describe eligible treatments included in the treatment network, and note whether any have been clustered or merged into the same node (with justification).*	5
Information sources	7	Describe all information sources (e.g., databases with dates of coverage, contact with study authors to identify additional studies) in the search and date last searched.	5
Search	8	Present full electronic search strategy for at least one database, including any limits used, such that it could be repeated.	5 and eTable1
Study selection	9	State the process for selecting studies (i.e., screening, eligibility, included in systematic review, and, if applicable,	5

		included in the meta-analysis).	
Data collection process	10	Describe method of data extraction from reports (e.g., piloted forms, independently, in duplicate) and any processes for obtaining and confirming data from investigators.	6
Data items	11	List and define all variables for which data were sought (e.g., PICOS, funding sources) and any assumptions and simplifications made.	5-6
**Geometry of the network**	**S1**	Describe methods used to explore the geometry of the treatment network under study and potential biases related to it. This should include how the evidence base has been graphically summarized for presentation, and what characteristics were compiled and used to describe the evidence base to readers.	eFigure3
Risk of bias within individual studies	12	Describe methods used for assessing risk of bias of individual studies (including specification of whether this was done at the study or outcome level), and how this information is to be used in any data synthesis.	5-6
Summary measures	13	State the principal summary measures (e.g., risk ratio, difference in means). *Also describe the use of additional summary measures assessed, such as treatment rankings and surface under the cumulative ranking curve (SUCRA) values, as well as modified approaches used to present summary findings from meta-analyses.*	6
Planned methods of analysis	14	Describe the methods of handling data and combining results of studies for each network meta-analysis. This should include, but not be limited to:<br>• *Handling of multi-arm trials;*<br>• *Selection of variance structure;*<br>• *Selection of prior distributions in Bayesian analyses; and*<br>• *Assessment of model fit.*	6
**Assessment of Inconsistency**	**S2**	Describe the statistical methods used to evaluate the agreement of direct and indirect evidence in the treatment network(s) studied. Describe efforts taken to address its presence when found.	6
Risk of bias across studies	15	Specify any assessment of risk of bias that may affect the cumulative evidence (e.g., publication bias, selective reporting within studies).	Not applicable
Additional analyses	16	Describe methods of additional analyses if done, indicating which were pre-specified. This may include, but not be limited to, the following:<br>• Sensitivity or subgroup analyses;<br>• Meta-regression analyses;<br>• *Alternative formulations of the treatment network; and*<br>• *Use of alternative prior distributions for Bayesian analyses (if applicable).*	6-9

**RESULTS†**			
Study selection	17	Give numbers of studies screened, assessed for eligibility, and included in the review, with reasons for exclusions at each stage, ideally with a flow diagram.	5, eFigure 1
**Presentation of network structure**	**S3**	Provide a network graph of the included studies to enable visualization of the geometry of the treatment network.	eFigure 3
**Summary of network geometry**	**S4**	Provide a brief overview of characteristics of the treatment network. This may include commentary on the abundance of trials and randomized patients for the different interventions and pairwise comparisons in the network, gaps of evidence in the treatment network, and potential biases reflected by the network structure.	Not applicable
Study characteristics	18	For each study, present characteristics for which data were extracted (e.g., study size, PICOS, follow-up period) and provide the citations.	9-10,eTable 4
Risk of bias within studies	19	Present data on risk of bias of each study and, if available, any outcome level assessment.	eFigure 2
Results of individual studies	20	For all outcomes considered (benefits or harms), present, for each study: 1) simple summary data for each intervention group, and 2) effect estimates and confidence intervals. *Modified approaches may be needed to deal with information from larger networks.*	10-12, Figure 1 and 2
Synthesis of results	21	Present results of each meta-analysis done, including confidence/credible intervals. *In larger networks, authors may focus on comparisons versus a particular comparator (e.g. placebo or standard care), with full findings presented in an appendix. League tables and forest plots may be considered to summarize pairwise comparisons.* If additional summary measures were explored (such as treatment rankings), these should also be presented.	10-12, Figure 1 and 2, eFigure 4, and eTable 7
**Exploration for inconsistency**	**S5**	Describe results from investigations of inconsistency. This may include such information as measures of model fit to compare consistency and inconsistency models, *P* values from statistical tests, or summary of inconsistency estimates from different parts of the treatment network.	Not applicable
Risk of bias across studies	22	Present results of any assessment of risk of bias across studies for the evidence base being studied.	Not applicable
Results of additional analyses	23	Give results of additional analyses, if done (e.g., sensitivity or subgroup analyses, meta-regression analyses*, alternative network geometries studied, alternative choice of prior distributions for Bayesian analyses,* and so forth).	Figure 1 and 2
**DISCUSSION**			
Summary of	24	Summarize the main findings, including the strength of	14

evidence		evidence for each main outcome; consider their relevance to key groups (e.g., healthcare providers, users, and policy-makers).	
Limitations	25	Discuss limitations at study and outcome level (e.g., risk of bias), and at review level (e.g., incomplete retrieval of identified research, reporting bias). *Comment on the validity of the assumptions, such as transitivity and consistency. Comment on any concerns regarding network geometry (e.g., avoidance of certain comparisons).*	17
Conclusions	26	Provide a general interpretation of the results in the context of other evidence, and implications for future research.	17-18
**FUNDING**			
Funding	27	Describe sources of funding for the systematic review and other support (e.g., supply of data); role of funders for the systematic review. This should also include information regarding whether funding has been received from manufacturers of treatments in the network and/or whether some of the authors are content experts with professional conflicts of interest that could affect use of treatments in the network.	18

Abbreviation: PICOS = population, intervention, comparators, outcomes, study design.

* Text in italics indicates wording specific to reporting of network meta-analyses that has been added to guidance from the PRISMA statement.
† Authors may wish to plan for use of appendices to present all relevant information in full detail for items in this section.

Reference:

Hutton B, Salanti G, Caldwell DM et al. The PRISMA extension statement for reporting of systematic reviews incorporating network meta-analyses of health care interventions: checklist and explanations. Ann Intern Med 2015; 162: 777-784.

eTable 3. CHEERS Checklist.

Section/item	Item No	Recommendation	Reported on page No
**Title and abstract**			
Title	1	Identify the study as an economic evaluation or use more specific terms such as "cost-effectiveness analysis", and describe the interventions compared.	1
Abstract	2	Provide a structured summary of objectives, perspective, setting, methods (including study design and inputs), results (including base case and uncertainty analyses), and conclusions.	2
**Introduction**			
Background and objectives	3	Provide an explicit statement of the broader context for the study.	3-4
		Present the study question and its relevance for health policy or practice decisions.	
**Methods**			
Target population and subgroups	4	Describe characteristics of the base case population and subgroups analysed, including why they were chosen.	7
Setting and location	5	State relevant aspects of the system(s) in which the decision(s) need(s) to be made.	7
Study perspective	6	Describe the perspective of the study and relate this to the costs being evaluated.	7
Comparators	7	Describe the interventions or strategies being compared and state why they were chosen.	7
Time horizon	8	State the time horizon(s) over which costs and consequences are being evaluated and say why appropriate.	7
Discount rate	9	Report the choice of discount rate(s) used for costs and outcomes and say why appropriate.	7
Choice of health outcomes	10	Describe what outcomes were used as the measure(s) of benefit in the evaluation and their relevance for the type of analysis performed.	7
Measurement of effectiveness	11a	*Single study-based estimates:* Describe fully the design features of the single effectiveness study and why the single study was a sufficient source of clinical effectiveness data.	6

	11b	*Synthesis-based estimates:* Describe fully the methods used for identification of included studies and synthesis of clinical effectiveness data.	
Measurement and valuation of preference based outcomes	12	If applicable, describe the population and methods used to elicit preferences for outcomes.	7-8
Estimating resources and costs	13a	*Single study-based economic evaluation:* Describe approaches used to estimate resource use associated with the alternative interventions. Describe primary or secondary research methods for valuing each resource item in terms of its unit cost. Describe any adjustments made to approximate to opportunity costs.	8-9
	13b	*Model-based economic evaluation:* Describe approaches and data sources used to estimate resource use associated with model health states. Describe primary or secondary research methods for valuing each resource item in terms of its unit cost. Describe any adjustments made to approximate to opportunity costs.	
Currency, price date, and conversion	14	Report the dates of the estimated resource quantities and unit costs. Describe methods for adjusting estimated unit costs to the year of reported costs if necessary. Describe methods for converting costs into a common currency base and the exchange rate.	8
Choice of model	15	Describe and give reasons for the specific type of decision-analytical model used. Providing a figure to show model structure is strongly recommended.	8 and eFigure 5
Assumptions	16	Describe all structural or other assumptions underpinning the decision-analytical model.	8
Analytical methods	17	Describe all analytical methods supporting the evaluation. This could include methods for dealing with skewed, missing, or censored data; extrapolation methods; methods for pooling data; approaches to validate or make adjustments (such as half cycle corrections) to a model; and methods for handling population heterogeneity and uncertainty.	8-9
**Results**			

Study parameters	18	Report the values, ranges, references, and, if used, probability distributions for all parameters. Report reasons or sources for distributions used to represent uncertainty where appropriate. Providing a table to show the input values is strongly recommended.	8 (Table 1)
Incremental costs and outcomes	19	For each intervention, report mean values for the main categories of estimated costs and outcomes of interest, as well as mean differences between the comparator groups. If applicable, report incremental cost-effectiveness ratios.	12 (Table 2)
Characterising uncertainty	20a	*Single study-based economic evaluation:* Describe the effects of sampling uncertainty for the estimated incremental cost and incremental effectiveness parameters, together with the impact of methodological assumptions (such as discount rate, study perspective).	13
	20b	*Model-based economic evaluation:* Describe the effects on the results of uncertainty for all input parameters, and uncertainty related to the structure of the model and assumptions.	
Characterising heterogeneity	21	If applicable, report differences in costs, outcomes, or cost-effectiveness that can be explained by variations between subgroups of patients with different baseline characteristics or other observed variability in effects that are not reducible by more information.	13
**Discussion**			
Study findings, limitations, generalisability, and current knowledge	22	Summarise key study findings and describe how they support the conclusions reached. Discuss limitations and the generalisability of the findings and how the findings fit with current knowledge.	15-16
**Other**			
Source of funding	23	Describe how the study was funded and the role of the funder in the identification, design, conduct, and reporting of the analysis. Describe other non-monetary sources of support.	18
Conflicts of interest	24	Describe any potential for conflict of interest of study contributors in accordance with journal policy. In the absence of a journal policy, we recommend authors comply with International Committee of Medical Journal Editors recommendations.	19

A good template page for CHEERS Checklist is as follows: http://www.ispor.org/TaskForces/EconomicPubGuidelines.asp.

Reference:

Husereau D, Drummond M, Petrou S, et al. Consolidated health economic evaluation reporting standards (CHEERS) — Explanation and elaboration: A report of the ISPOR health economic evaluations publication guidelines good reporting practices task force. Value Health 2013;16:231-50.

eTable 4. List of the studies included in the meta-analysis.

Study	Trial name or ID	Total sample size	Intervetion	Sample size	Control	Sample size	Median OS (months)	HR for OS (95% CI)	Median PFS (months)	HR for PFS (95% CI)	Patients with grade 3 or higher AEs, n (%)
Reck, 2016	CA184-156	1132	Ipilimumab plus Chemotherapy	478	Chemotherapy	476	11.0 vs 10.9	0.94 (0.81 to 1.09)	4.6 vs 4.4	0.85 (0.75 to 0.97)	48.3 vs 45.0
Rudin, 2020	KEYNOTE-604	453	Pembrolizumab plus Chemotherapy	228	Chemotherapy	225	10.8 vs 9.7	0.78 (0.63 to 0.97)	4.5 vs 4.3	0.75 (0.61 to 0.91)	83.0 vs 80.3
Goldman,2020	CASPIAN	805	Durvalumab plus Chemotherapy	268	Chemotherapy	269	12.9 vs 10.5	0.75 (0.62 to 0.91)	5.1 vs 5.4	0.80 (0.66 to 0.96)	64.5 vs 65.0
Goldman,2020	CASPIAN	805	Durvalumab with tremelimumab plus Chemotherapy	268	Chemotherapy	269	10.4 vs 10.5	0.82 (0.68 to 1.00)	4.9 vs 5.4	0.84 (0.70 to 1.01)	73.7 vs 65.0
Horn, 2021	IMpower133	403	Atezolizumab plus Chemotherapy	201	Chemotherapy	202	12.3 vs 10.3	0.76 (0.60 to 0.95)	5.2 vs 4.3	0.77 (0.63 to 0.95)	58.6 vs 57.7

Abbreviation: ID, Identification; OS ,Overall Survival; PFS, progression-freesurvival; CI, confidence interval; AEs, Adverse Events.

eTable 5. Drug dose and cost.

Drug	Dose	Infusion Timing	Unit costs(＄)
Atezolizumab plus Chemotherapy	atezolizumab,1200mg;	four 3-week cycles; followed by maintenance atezolizumab every 3 weeks	7.9749 per 1mg
	etoposide, 100mg/m^2;		0.0795 per 1mg
	carboplatin, AUC 5.0mg/ml/min		0.05286 per 1mg
Durvaluma plus Chemotherapy	durvalumab,1500mg;	four 3-week cycles; followed by maintenance durvalumab every 4 weeks	7.9749 per 1 mg
	etoposide, 100mg/m^2;		0.0795 per 1mg
	carboplatin, AUC 5.0mg/ml/min		0.05286 per 1mg
Pembrolizumab plus Chemotherapy	pembrolizumab, 200 mg;	four 3-week cycles; followed by maintenance pembrolizumab every 3 weeks	52.754 per 1 mg
	etoposide, 100mg/m^2;		0.0795 per 1mg
	carboplatin, AUC 5.0mg/ml/min		0.05286 per 1mg
Ipilimumab plus Chemotherapy	Ipilimumab, 10 mg/kg;	four 3-week cycles; followed by maintenance ipilimumab every 3 weeks	158.648 per 1mg
	etoposide, 100mg/m^2;		0.0795 per 1mg
	carboplatin, AUC 5.0mg/ml/min		0.05286 per 1mg
Chemotherapy	etoposide, 100mg/m^2;	four 3-week cycles	0.0795 per 1mg
	carboplatin, AUC 5.0mg/ml/min		0.05286 per 1mg
Topotecan	Topotecan, 1mg/m^2	d1-5	7.66 per 1 mg

eTable 6. Summary of statistical goodness-of-fit of K-M curve.

	Exponential	Weibull	Gompertz	Log-logistic	Lognormal
**Atezolizumab plus Chemotherapy OS curve**					
AIC	39.35	37.90	38.29	37.00	36.99
BIC	40.35	39.89	40.28	38.99	38.98
**Durvaluma plus Chemotherapy OS curve**					
AIC	39.95	37.64	37.82	36.87	36.83
BIC	40.95	39.63	39.82	38.87	38.82
**Pembrolizumab plus Chemotherapy OS curve**					
AIC	45.40	43.78	43.80	42.97	42.93
BIC	46.45	45.87	45.99	45.06	45.02
**Ipilimumab plus Chemotherapy OS curve**					
AIC	62.69	56.27	62.63	51.20	51.80
BIC	63.69	58.26	64.62	53.19	53.80
**Chemotherapy OS curve**					
AIC	70.58	69.21	79.53	61.06	62.25
BIC	71.62	71.30	81.63	63.15	64.35
**Atezolizumab plus Chemotherapy PFS curve**					
AIC	63.49	62.81	63.13	55.63	56.67
BIC	64.20	64.22	64.55	57.05	58.09
**Durvaluma plus Chemotherapy PFS curve**					
AIC	83.61	81.13	85.97	75.90	76.72
BIC	84.66	83.22	88.06	77.98	78.81
**Pembrolizumab plus Chemotherapy PFS curve**					
AIC	55.36	51.10	51.15	45.34	46.12
BIC	55.92	52.23	52.28	46.47	47.25
**Ipilimumab plus Chemotherapy PFS curve**					
AIC	148.67	120.70	647.42	67.75	74.79
BIC	149.31	121.97	648.70	69.03	76.07
**Chemotherapy PFS curve**					
AIC	145.69	106.45	433.27	62.06	68.67
BIC	146.26	107.58	434.40	63.19	69.80

Abbreviation: OS, overall survival; PFS, progression-free survival; AIC, Akaike's information criterion; BIC, Bayesian information criterion.

As for the curves listed in the table, the lognormal and log-logistic distribution had the lowest AIC and BIC. However, the Log-logistic and lognormal models can incorporate non-monotonic hazards but typically have long tails due to a reducing hazard as time increases after a certain point. Actually, the visual fits of the curves (eFigure 6 and 7 in the supplementary material) showed that lognormal and log-logistic distribution extended tail, which would likely overestimate OS and PFS in the long term based on clinical experts' opinion.

Weibull distributions are flexible and widely used were matched to the number of patients in the three states over time, as its can monotonically increase or decrease the hazard function, it is suitable for estimating the event that occurs in the early follow-up work period. Therefore, the Weibull distributions was likely to be the most reasonable parametric survival model.

eTable 7. safety profiles.

**AC**	1.04 (0.26 to 4.20)	2.63 (0.45 to 15.33)	1.31 (0.34 to 5.16)	0.92 (0.38 to 2.22)	0.64 (0.28 to 1.47)	0.52
0.97 (0.57 to 1.65)	**DC**	2.54 (0.49 to 13.20)	1.27 (0.26 to 6.11)	0.89 (0.28 to 2.87)	0.62 (0.20 to 1.92)	0.52
1.15 (0.67 to 1.95)	1.18 (0.72 to 1.93)	**DTC**	0.50 (0.08 to 3.35)	0.35 (0.07 to 1.72)	0.24 (0.05 to 1.16)	0.13
1.08 (0.62 to 1.88)	1.11 (0.66 to 1.86)	0.94 (0.56 to 1.58)	**PC**	0.70 (0.23 to 2.17)	0.49 (0.16 to 1.45)	0.37
1.09 (0.68 to 1.75)	1.13 (0.73 to 1.73)	0.95 (0.62 to 1.47)	1.01 (0.64 to 1.61)	**IC**	**0.70 (0.51 to 0.96)**	0.56
0.96 (0.65 to 1.43)	0.99 (0.70 to 1.40)	0.84 (0.59 to 1.19)	0.89 (0.61 to 1.31)	0.88 (0.68 to 1.14)	**C**	0.90
0.63	0.57	0.31	0.43	0.38	0.70	**P-Score**

Abbreviation: AC; atezolizumab plus chemotherapy; DC; durvaluma plus chemotherapy; DTC; durvalumab with tremelimumab plus chemotherapy; PC; pembrolizumab plus chemotherapy; IC; ipilimumab plus chemotherapy; C; chemotherapy.

The pooled ORs; 95%CI and P-values for all-grade treatment-related adverse events (upper triangle) and grade 3–4 treatment-related adverse events (lower triangle) of the network meta-analysis; significant results are in bold.

eTable 8. Pairwise comparison of ICER ($/QALY).

**Overall population**					
**Treatment**	Chemotherapy	Atezolizumab plus Chemotherapy	Durvaluma plus Chemotherapy	Pembrolizumab plus Chemotherapy	Ipilimumab plus Chemotherapy
Chemotherapy	NA	321,452	399,978	675,358	5,437,894
Atezolizumab plus Chemotherapy	321,452	NA	Dominated	Dominated	Dominated
Durvaluma plus Chemotherapy	399,978	Dominated	NA	Dominated	Dominated
Pembrolizumab plus Chemotherapy	675,358	Dominated	Dominated	NA	Dominated
Ipilimumab plus Chemotherapy	5,437,894	Dominated	Dominated	Dominated	NA
**Population with brain metastases**					
**Treatment**	Chemotherapy	Atezolizumab plus Chemotherapy	Durvaluma plus Chemotherapy	Pembrolizumab plus Chemotherapy	Ipilimumab plus Chemotherapy
Chemotherapy	NA	1,196,550	621,350	Dominated	Dominated
Atezolizumab plus Chemotherapy	1,196,550	NA	333,750	Dominated	Dominated[a]

Durvaluma plus Chemotherapy	621,350	333,750	NA	832,748	Dominated
Pembrolizumab plus Chemotherapy	Dominated	Dominated	832,748	NA	Dominated
Ipilimumab plus Chemotherapy	Dominated	Dominated	Dominated	Dominated	NA

Population with non-brain metastases

**Treatment**	Chemotherapy	Atezolizumab plus Chemotherapy	Durvaluma plus Chemotherapy	Pembrolizumab plus Chemotherapy	Ipilimumab plus Chemotherapy
Chemotherapy	NA	429,606	718,640	1,272,538	20,322,400
Atezolizumab plus Chemotherapy	429,606	NA	Dominated	Dominated	Dominated
Durvaluma plus Chemotherapy	718,640	Dominated	NA	Dominated	Dominated
Pembrolizumab plus Chemotherapy	1,272,538	Dominated	Dominated	NA	Dominated
Ipilimumab plus Chemotherapy	20,322,400	Dominated	Dominated	Dominated	NA

Abbreviation: ICER, incremental cost-effectiveness ratio; LY, life-year; QALY, quality-adjusted life-year.

Dominated: Treatment showed lower effectiveness and higher cost, as compared with another treatment.
